# New Approach to the Systematics of the Section *Psammiris* (*Iris*, Iridaceae): What Does Chloroplast DNA Sequence Tell Us?

**DOI:** 10.3390/plants12061254

**Published:** 2023-03-09

**Authors:** Eugeny V. Boltenkov, Elena V. Artyukova

**Affiliations:** 1 Botanical Garden-Institute, Far Eastern Branch, Russian Academy of Sciences, Vladivostok 690024, Russia; 2 Federal Scientific Center of the East Asia Terrestrial Biodiversity, Far Eastern Branch, Russian Academy of Sciences, Vladivostok 690022, Russia

**Keywords:** classification, *Iris*, molecular phylogeny, morphology, nomenclature, *Psammiris*, taxonomy, typification

## Abstract

*Iris* sect. *Psammiris* comprises rhizomatous perennials distributed in the north temperate zone of Eurasia. The systematics of the section are currently based on morphology, and the phylogenetic relationships within it still remain unclear. In the framework of *Iris* systematics, we conducted molecular and morphological analyses of the currently recognized *I*. sect. *Psammiris* species to elucidate the taxonomic composition and relationships within the section. The phylogenetic reconstructions based on sequence variation of four noncoding chloroplast DNA regions support the monophyly of *I*. sect. *Psammiris*, which includes *I. tigridia*, while *I. potaninii* var. *ionantha* belongs to *I.* sect. *Pseudoregelia*. The proposed novel classification of *I*. sect. *Psammiris* recognizes three series: an autonymic series with *I. humilis*, *I. bloudowii*, and I*. vorobievii* and two unispecific series (*I*. ser. *Potaninia* with *I. potaninii* and *I*. ser. *Tigridiae* with *I. tigridia*). In addition, the taxonomic statuses of *I. arenaria*, *I. ivanovae*, *I. kamelinii*, *I. mandshurica*, *I. pineticola*, *I. psammocola*, and *I. schmakovii* are clarified herein. We provide a revised taxonomic treatment for *I*. sect. *Psammiris*, including notes on the types; updated information on species synonymy, distributions, habitats, and chromosome numbers; and a new identification key to the species. Three lectotypes are designated here.

## 1. Introduction

Psammirises, or sand irises, are a small group of *Iris* L. (Iridaceae) that comprises rhizomatous perennials distributed exclusively in the north temperate zone of Eurasia and found in sandy soils of steppes, open meadows, and hillsides. As the most cold-resistant among *I*. subgen. *Iris* (bearded irises), psammirises have always attracted attention as garden plants [1]. In fact, almost all psammirises are successfully blooming and fruiting in culture in Yakutsk (at approximately latitude 62° N), Russia [2].

**Figure 1:**
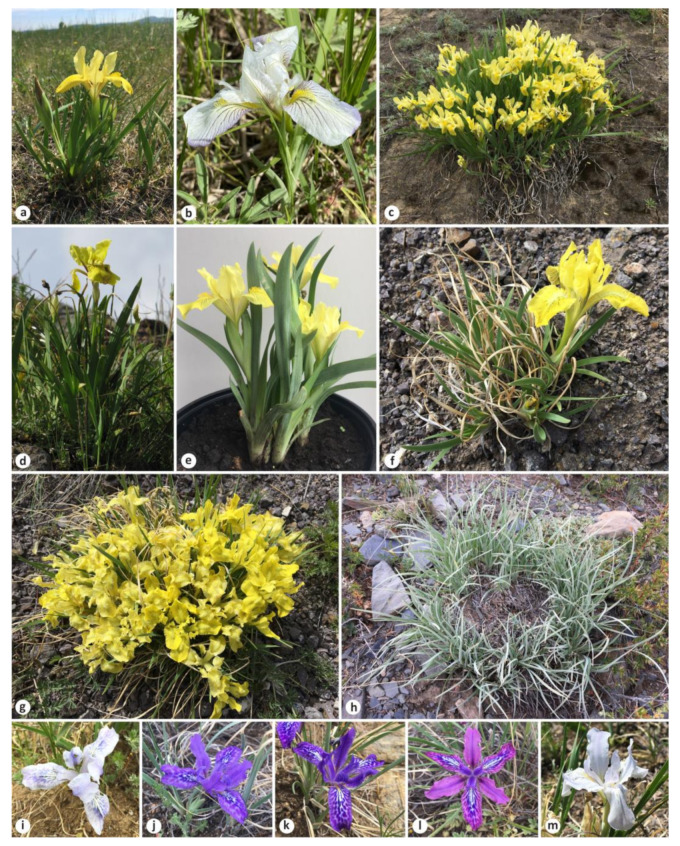
The species of *Iris* sect. *Psammiris*: (**a**) *I. humilis*, habit (Russia, Zabaykalsky Krai, vicinities of Ingoda Village); (**b**) *I. humilis*, a white-flowered form (Russia, Primorsky Krai, Khankaysky District); (**c**) *I. humilis*, in clump (Russia, Republic of Buryatia, Tarbagataysky District); (**d**) *I. bloudowii*, habit (Kazakhstan, Almaty Region, Dzhungraian Alatau); (**e**) *I. vorobievii*, habit (Russia, Primorsky Krai, vicinities of Kraskino); (**f**,**g**) *I. potaninii*, habit (Russia, Buryatia, vicinities of Novoselenginsk); (**h**) *I. potaninii*, in clump (Russia, Altai Republic, Kosh-Agach District, vicinities of Verkhniye Boguty Lake); (**i**–**l**) *I. tigridia*, flower color (Russia, Republic of Buryatia, Kyakhtinsky District, shtab-lekarskaya zaimka); (**m**) *I. tigridia*, a white-flowered form (Russia, Zabaykalsky Krai, vicinities of Khara-Byrka); (**a**,**c**,**e**–**m**) by E. Boltenkov, (**b**) by A. Malyk, (**d**) by A. Grebenjuk.

This group was established by Spach [3] as *I*. subgen. *Psammiris* Spach for plants with helically twisted withering flowers and arillate seeds; however, Baker [4] transferred it to *I.* sect. *Pogoniris* (Spach) Baker, which was supported in references [5,6,7]. Lawrence [8] placed some of psammirises in *I*. ser. *Pumilae* G.H.M.Lawr. and others in *I.* subsect. *Hexapogon* (Bunge) Benth. Taylor [9] eventually re-established psammirises at the section level, which was subsequently supported by the authors of [1,10,11,12,13,14,15,16]. Four species were included in *I*. sect. *Psammiris* (Spach) J.J.Taylor [9]: *I. humilis* Georgi, *I. bloudowii* Ledeb., *I. mandshurica* Maxim., and *I. potaninii* Maxim. (Figure 1). Currently, the section comprises eight species including the recently described *I. vorobievii* N.S.Pavlova, *I. psammocola* Y.T.Zhao, *I. kamelinii* Alexeeva, and *I. schmakovii* Alexeeva [15,17]. Despite this recent advancement in the knowledge of the section, there are still numerous uncertainties regarding its taxonomic composition and systematics.

One of the best-known species in the group, *I. humilis* (Figure 1a–c), was initially described by Johann Gottlieb Georgi in an area of the Lake Baikal coast between the Angara River and Olkhon Island [18]. Then, for about two centuries, it was referred to by various researchers, including Georgi [19], as *I. flavissima* Pall. [5,6,7,20,21,22,23,24,25,26,27], which had been described on the basis of plants from Transbaikalia [28]. Bobrov [29] noted that *I. humilis* and *I. flavissima* were actually the same species and that the name *I. humilis* had priority, as was later supported (e.g., [9,30]). However, the taxonomic concept within *I. humilis* has varied widely.

Another species, *I. mandshurica*, was described based on plants collected near the Razdolnaya River (Primorsky Krai, Russia). It was often considered an ally of *I. flavissima* or as an intermediate between *I. flavissima* and *I. bloudowii* [5,24,26,31]. In the *Iris* treatments of the Russian Far East flora, *I. mandshurica* was indicated as a synonym of *I. humilis* [32,33]. Nevertheless, a preliminary molecular analysis shows that *I. mandshurica* merits to be recognized as a separate species [34].

After being described, *I. arenaria* Waldst. et Kit. was considered a Hungarian representative of *I. humilis* (=*I. flavissima*) [10,13,24,26,35] or, less commonly, as a separate species [5,36,37,38]. Ugrinsky concluded that *I. flavissima* and *I. arenaria* are forms of a single species that differ mainly in geographical terms: *I. flavissima* subsp. *stolonifera* f. *occidentalis* Ugr. (=*I. arenaria*) is a western representative from Eastern Europe, and *I. flavissima* subsp. *stolonifera* f. *orientalis* Ugr. is an eastern representative from Ukraine and western Russia [22,23]. Klokov recognized the latter form at the species level under the name *I. pineticola* Klokov [39], which is currently included in the species aggregate with *I. humilis* [1,10,12,13,26,40] or is considered as endemic to Ukraine [41]. In addition, *I. schmakovii*, originally described as *I. humilis* var. *umbrosa* Alexeeva from Mongolia [17], is currently treated as a synonym of *I. humilis* [42].

*Iris bloudowii* (Figure 1d) was described by Carl Friedrich von Ledebour [43] from plants gathered by him in the Gromotukha River valley on the southern forested slope of the Ivanovsky Ridge (Kazakhstan Altai) and originally identified as *I. flavissima* [44] (p. 91). It is closely related to *I. humilis* and was therefore treated as a larger variety of *I. flavissima* [5,21,26] or a synonym of *I. humilis* [45]. *Iris bloudowii* was also regarded as a separate species [5,20,23,24], which is supported to date [15,27,46,47,48].

While revising the Far Eastern Iridaceae, Nonna Pavlova [32] came to the conclusion that the plants from southern Khasansky District (Primorsky Krai, Russia) collected near the borders with China and North Korea were a separate species, *I. vorobievii* (Figure 1e). It is a little-known species that was referred to as *I. mandshurica* by Georgi Rodionenko [1,12]. He believed that *I. vorobievii* is unrelated to *I. humilis* and should be transferred to *I*. sect. *Pseudoregelia* Dykes, which has not been confirmed by a preliminary molecular study [34].

The well-known psammiris, *I. potaninii* (Figure 1f–h), was described by Carl Johann Maximowicz from Russia (Altai and Transbaikalia) and Mongolia [20]. Rodionenko suggested *I. potaninii* to be transferred to *I*. sect. *Pseudoregelia*, since it is the closest relative to *I. tigridia* Bunge [1]. Moreover, phylogenetic studies showed that *I. potaninii* is not monophyletic, as an autonymic variety belongs to *I.* sect. *Psammiris*, whereas *I. potaninii* var. *ionantha* Y.T.Zhao described from Qinghai Province, China [49], is in *I.* sect. *Pseudoregelia* [14,50,51]. It was stated that *I. potaninii* var. *ionantha* is actually the same taxon as *I*. *thoroldii* Baker [52], which was previously recognized as a distinct species [27,47]. Despite this statement, *I. potaninii* var. *ionantha* has been recognized at the species level as *I. zhaoana* M.B.Crespo, Alexeeva et Y.E.Xiao and included in *I.* sect. *Pseudoregelia* ser. *Tigridiae* Doronkin [16].

*Iris psammocola*, a little-known species, was described by Yu-Tang Zhao on the basis of a single specimen collected in the vicinity of Baijiatan, Ningxia Hui Autonomous Region, China [53]. It has been accepted in the botany databases [42,54]. In 2005, *I. psammocola* was reported, along with *I. potaninii*, from the Tsugeer-Els area, a sand cluster of the Ubsunorskaya Kotlovina Biosphere Reserve, southeastern Republic of Tuva, Russia [55]. In addition, *I. potaninii* var. *arenaria* Doronkin, described from Kyakhtinsky District, southern Republic of Buryatia, Russia [11], was synonymized with *I. psammocola* [55]. Subsequently, *I. psammocola* was reported from the Altan-Els sand dune region of the Borig-Del-Els sandy areas, the Mongolian part of the Uvs Lake Basin [56]. It was asserted that *I. psammocola* occurs only on sandy arrays and has a disjunctive distribution range in Central Asia [55,56]. It is a relative of *I. potaninii* and has a chromosome number of 2*n* = 22 [55]. The same chromosome number has been reported for *I. potaninii* from eleven localities in the Altai Republic [57,58], from the Republic of Buryatia [57,59], from the Republic of Tuva, and the Zabaykalsky Krai [60]. In our opinion, the Russian populations of *I. psammocola* remain in question and require further studies, since their identity to *I. potaninii* is probable.

*Iris kamelinii*, another relative of *I. potaninii*, was described by Nina Alexeeva on the basis of plants collected near Verkhniye Boguty Lake on the western side of the Chikhachev Ridge, Southeast Altai Mountains, Russia [61]. According to the author, *I. kamelinii* in the type locality occurs together with *I. potaninii* [15,62] and has the same chromosome number, 2*n* = 22 [58,61]. Moreover, *I. kamelinii* shows the nearest affinity with *I. potaninii* in flowering [63] (p. 51) and in seed morphology [64]. Meanwhile, *I. kamelinii*, treated as endemic to the Altai Mountain Country, also grows in Mongolia and China [17,65].

Phylogenetic analyses of the *Iris* species based on cpDNA sequence data have shown that *I*. sect. *Psammiris* and *I*. sect. *Pseudoregelia* are not monophyletic [14,50,51,66]. As most authors suggested, *I. tigridia* (Figure 1i–m) has been included in *I*. sect. *Pseudoregelia* [1,10,11,12,30,67,68]. Accordingly, the combination *I*. sect. *Pseudoregelia* ser. *Tigridiae* has been proposed for *I. tigridia* and its relative, *I. ivanovae* Doronkin [11]. However, some authors consider *I. tigridia* and *I. potaninii* (from *I.* sect. *Psammiris*) to be closely related [1,27]. In a phylogenetic study of Siberian irises, *I. tigridia* and *I. ivanovae* formed a sister group with psammirises [69]. Other molecular studies also supported the inclusion of *I. tigridia* in *I.* sect. *Psammiris* [14,50,51,66]. Subsequently, however, the specimen cited as “… *R01-18*” in references [50,66] was interpreted as a misidentification with *I. tigridia* and referred to as *I. kamelinii* [15]. Nevertheless, a recent study confirmed that *I.* sect. *Psammiris* is a monophyletic taxon when *I. tigridia* is included [14]. Despite the considerations mentioned above, it was stated that *I. tigridia* and *I. ivanovae*, both with single-flowered stems, belong to *I.* sect. *Caespitosae* Alexeeva [15,17,70]. However, *I. pandurata* Maxim., the type species of *I.* sect. *Caespitosae* [71], is attributed to *I*. sect. *Pseudoregelia* [68,72]. It is a distinct species characterized by a two-flowered stem and is narrowly endemic to China, distributed in Gansu and Qinghai provinces [27,47,68].

*Iris ivanovae*, described from Kharanor, Zabaykalsky Krai, Russia, is considered to be a Transbaikalian, Mongolian, and Chinese representative of *I. tigridia* [73]. All experts on the Mongolian flora listed *I. tigridia* for the Khuvsgul, Khentei, Khangai, Mongolian Dahuria, and Middle Khalkha phytogeographical regions [27,74,75,76,77]. Moreover, Gubanov regarded *I. ivanovae* as a synonym of *I. tigridia* [76]. On the contrary, it was stated that all the plants from Mongolia previously named *I. tigridia* [25,27,76] belong to *I. ivanovae* [17,70,78]. Additionally, *I. ivanovae* is not accepted by the authors of the *Flora of China* since they did not see any specimens [46].

In view of all the above facts, a molecular study would be a great contribution to understanding the taxonomic composition and phylogenetic relationships among the *I.* sect. *Psammiris* species. A few studies based on cpDNA data have examined the relationships between different taxa within *Iris*, including psammirises [14,50,51,66,67,69,72], and elucidated the *I.* sect. *Psammiris* systematics, although only to a limited extent. A combination of *trnH-psbA* and *trnL-trnF* was previously proposed as the core barcode for plants [79]. As in our previous publications, in the present study, we focused on nucleotide sequences of four cpDNA noncoding regions (*trnH-psbA*, *rps4-trnS*^GGA^, *trnS-trnG*, and *trnL-trnF*) that proved to be useful as phylogenetic markers [34] and that we widely applied to assess interspecific relationships in *I*. subgen. *Limniris* (Tausch) Spach [80,81,82]. In the framework of the taxonomic research carried out on *Iris*, the objectives of the present study are as follows: (1) clarify the phylogenetic relationships of *I*. sect. *Psammiris* and *I. potaninii* var. *ionantha* with *I. tigridia* using four cpDNA regions; (2) elucidate the phylogenetic relationships within *I*. sect. *Psammiris* and determine the taxonomic statuses of *I. arenaria*, *I. ivanovae*, *I. kamelinii*, *I. mandshurica*, *I. pineticola*, *I. psammocola*, and *I. schmakovii*; (3) study the morphological characters of the *I*. sect. *Psammiris* species; and (4) compare the results of molecular and morphological studies in order to resolve the systematics of *I.* sect. *Psammiris*.

## 2. Materials and Methods

### 2.1. Taxa Used

**Figure 2:**
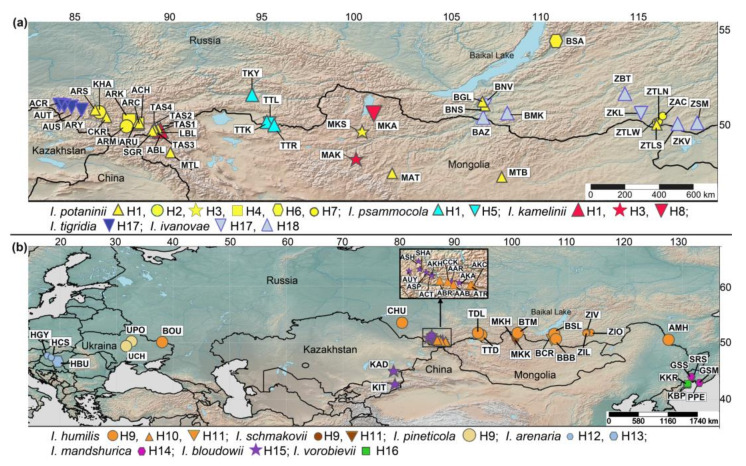
Map showing the geographical origin of the *Iris* samples analyzed in the present study (composed using https://www.simplemappr.net, CC 1.0; accessed on 4 October 2022) and illustrating the distribution of cpDNA haplotypes: (**a**) the *I*. sect. *Psammiris* species, *I. tigridia*, and *I. ivanovae*; (**b**) the *I*. sect. *Psammiris* species. For locality and haplotype codes, see Table 1.

We attempted to provide an extensive taxon sampling as possible and ensure that all accessions were fully verified. One of us (E.V. Boltenkov) undertook two botanical expeditions to southern Siberia (Russia): to the Altay Republic in 2020 and to Transbaikalia (Republic of Buryatia and Zabaykalsky Krai) in 2021. In addition, we collected plant material in Primorsky Krai, Russia, in 2020–2021. The complete list of the examined taxa, including information on samples, is provided in Table 1. The collected samples approximately represent the distribution range of the *I.* sect. *Psammiris* species (Figure 2).

The taxon samples for the present study are as follows: *I. psammocola* from the Republic of Tuva, Russia, including the sample TTL specified in reference [55] (four accessions); *I. potaninii* from the Altai Republic, Republic of Buryatia, as well as Zabaykalsky Krai, Russia, and Mongolia (25 accessions); *I. kamelinii* from the type locality (ABL) and two of the three Mongolian specimens specified in reference [17] (three accessions); *I. bloudowii* from Kyrgyzstan, Kazakhstan, and the Altai Republic, Russia, including AUY, a sample closest to the type locality (10 accessions); *I. pineticola* from a pine forest in Ukraine west of the type locality (two accessions); *I. humilis* from Belgorod Oblast, Altai Krai, as well as the republics of Altai, Tuva, and Buryatia, Zabaykalsky Krai, and Amur Oblast, Russia, including four samples from the type locality of *I. flavissima* (17 accessions); Hungarian samples of *I. arenaria* from the location where the species was described (three accessions); *I. schmakovii* from the type locality (two accessions); *I. mandshurica* from Primorsky Krai, Russia, including two samples (GSS and SRS) from the type locality (four accessions); *I. vorobievii* from Primorsky Krai, Russia, including a sample (KKR) from the type locality (two accessions); *I. tigridia* from the Altai Republic, including a sample (ACR) from the type locality (four accessions); *I. ivanovae* from the Republic of Buryatia and Zabaykalsky Krai, including a sample (ZKV) from the type locality (seven accessions); *I. goniocarpa* Baker from Sichuan and Gansu provinces, China (two accessions); and *I. potaninii* var. *ionantha* from Qinghai Province, China (one accession). The sampling localities for each species under study (except the *I.* sect. *Pseudoregelia* species) are shown in Figure 2. Two samples (CQM and CSJ) for which accurate species identification by morphological features was impossible were labeled as unidentified *Iris* samples.

**Figure 3:**
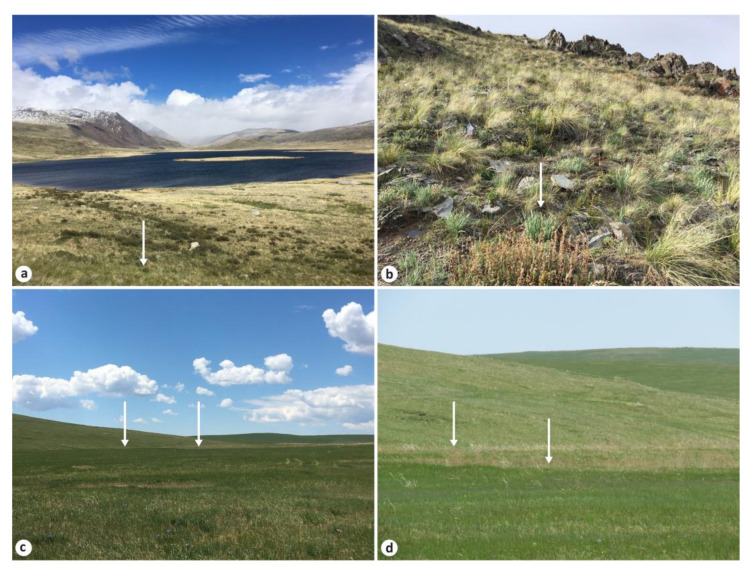
The type localities of irises are as follows: (**a**,**b**) *I. kamelinii* (Russia, Altai Republic, Kosh-Agach District, vicinities of Verkhniye Boguty Lake); (**c**,**d**) *I. ivanovae* (Russia, Zabaykalsky Krai, vicinities of Kharanor Village, together with *Stipa krylovii*); (**a**–**c**) by E. Boltenkov, (**d**) by D. Sandanov. Arrows indicate locations of irises.

During the fieldwork in the type localities, *I. kamelinii* was collected on 6 June 2020 from the northern slope opposite the northern bank of Verkhniye Boguty Lake, where it was found in flowering on soddy soils of mountainous steppes [84] on the hill and in fruiting opposite the hill at the base of the mountain slope (Figure 3a,b). *Iris ivanovae* was collected on 5 June 2021 at the end of flowering from a chestnut soil in a dry steppe heated at noon, where it was found growing, along with *Stipa krylovii* Roshev., on a sunlit lower part of the northern slope (Figure 3c,d). The type locality of *I. potaninii* var. *arenaria* was inspected twice; however, these plants were not found (although *I. tigridia* was abundant there), and the taxon is therefore not included in the analysis. No samples of *I. psammocola* were available from the type locality.

### 2.2. Plant Samples, DNA Extraction, and Sequencing

For genetic analysis, leaf samples were collected across the distribution range of the *I*. sect. *Psammiris* species. Total genomic DNA was isolated from the leaf samples collected during the fieldwork and dried in silica gel or taken from the herbarium specimens deposited at ALTB, E, KW, LE, MHA, MW, NENU, and UUH (herbarium codes according to *Index Herbariorum* [83]). The methods for DNA extraction, amplification, and direct sequencing of four cpDNA noncoding regions (*trnH-psbA*, *rps4-trnS*^GGA^, *trnS-trnG*, and *trnL-trnF*) were described previously [34,85]. The cycle sequencing reactions were performed on both strands, and fragments were separated on an ABI 3130 genetic analyzer (Applied Biosystems, Bedford, MA, USA) at the Joint Center of Biotechnology and Gene Engineering, the Federal Scientific Center of the East Asia Terrestrial Biodiversity, Far Eastern Branch, Russian Academy of Sciences (Vladivostok, Russia). Forward and reverse sequences for each region were assembled using the Staden Package, version 1.4 [86]. In a preliminary study, no polymorphism in the cpDNA regions was found in the sample of five individuals from the localities of *I. arenaria*, *I. humilis*, *I. kamelinii*, and *I. potaninii*; therefore, one specimen from each locality was used for further analysis. The sequences of the four cpDNA regions obtained for 88 accessions representing 14 taxa were deposited in the GenBank database. The accession numbers for all the sequences used are listed in Table 1.

### 2.3. Sequence Alignment and Phylogenetic Analysis

The sequences of each cpDNA region were aligned manually in SeaView version 4 [87] using the CLUSTAL algorithm, manually edited when necessary, and concatenated for each specimen. We included indels and length variation in mononucleotide repeats in the dataset because the repeatability tests allowed for exclusion of PCR errors. In the dataset, we also included the sequences for the most frequent haplotypes identified previously [34] in the localities of *I. humilis* (ALT-03), *I. mandshurica* (NAKH-01 and NAKH-07), and *I*. *vorobievii* (KRAS-01, KRAS-03, and KRAS-04). The haplotypes were identified on the basis of combined DNA sequences using DnaSP version 5 [88]. A network of haplotypes was constructed using Network version 4.6 [89], with each deletion/insertion treated as a single mutational event, regardless of size, and using the MJ method with default settings.

Phylogenetic relationships among the *I.* ser. *Psammiris* species were assessed using the MP and ML methods as implemented in PAUP version 4.0 b10 [90], as well as the BI method in MrBayes version 3.2.2 [91] via the CIPRES portal [92]. The dataset for the phylogenetic analysis included haplotypes obtained previously [80,81,82] for *I. dichotoma* of *I.* subgen. *Pardanthopsis* (Hance) Baker and for 14 species representing four series of *I.* subgen. *Limniris* as outgroups. For the MP method, optimal trees were found using a heuristic search with 1000 random addition sequence replicates, starting trees obtained via stepwise addition, TBR branch swapping, and the MulTrees option in effect. For the ML and BI methods, the GTR + I + G model was selected according to the Akaike information criterion using Modeltest version 3.6 [93]. ML heuristic searches were performed using the resulting model settings, 100 replicates of random sequence addition, TBR branch swapping, and the MULTrees option. In BI, using the default prior settings, two parallel MCMC runs were carried out for 10 million generations, with sampling every 1000 generations for a total of 10,000 samples. Convergence of the two chains was assessed, and PP was calculated from the trees sampled during the stationary phase. The robustness of nodes in the ML and MP trees was tested using bootstrap with 1000 replicates.

Degrees of divergence between the species were calculated based on nucleotide substitutions using DnaSP. Pairwise *F*_ST_ among them were determined by AMOVA as implemented in Arlequin version 3.5 [94]. Significance of genetic distances was tested using 1000 random permutations.

### 2.4. Morphological Data

To compile a morphological key to the accepted species of *I.* ser. *Psammiris* in the present study, 22 characters were selected for comparison: (1) rhizome shape (creeping, forming branches like stolons or shortened or nodose, slowly creeping (compact)), (2) rhizome diameter, (3) root shape (adventitious roots gradually tapering to the apex, not thickening (equal); fleshy at the proximal part, resembling a cone (obconical); or evenly thickened at the proximal part with wrinkled transverse patterns (contractile)), (4) root diameter (measured at the proximal end), (5) leaf shape (straight, sword-shaped rosette leaves with more or less parallel margins (ensiform) or one slightly convex margin falcate at the distal part and margins abruptly apically narrowed (subfalcate)), (6) leaf apex (rosette leaves apex straight or slightly incurved, gradually narrowed (narrowly acute), or abruptly narrowed (acute)), (7) leaf texture (rosette leaves noticeably tough or less tough and flexible (thin); the surfaces finely ribbed (smooth); or with discrete central veins (ribbed)), (8) leaf length (measured from the base to the apex of the longest rosette leaf), (9) leaf width (measured at the broadest part of the widest rosette leaf), (10) stem height (measured from the base of the flowering stem to the base of the outer bract), (11) stem branching (classified as simple, bearing only the terminal cluster (designated as 0), or branched, with 1–2 lateral one-flowered cluster(s)), (12) number of flowers (flowers per stem), (13) cauline leaf length (measured from the base to the apex of the upper cauline leaf), (14) number of bracteoles (secondary bracts, i.e., bracteoles, per terminal cluster of the inflorescence), (15) bract length (measured from the base to the apex of the outer bract of the terminal cluster), (16) bract texture (coriaceus, pliable but thin when dry bracts (tough) or membranous and somewhat translucent (thin)), (17) pedicel length (measured from the base of the terminal cluster to the ovary base of the first blooming flower), (18) tube length (measured from the ovary apex to the base of the outer perianth segments, i.e., falls), (19) flower color (the flower color based on personal observations), (20) fruit length and (21) width (obtained for the first fruit of the terminal cluster), and (22) fruit shape.

The scores of the characters for each species were obtained from living specimens collected from wild localities; from our own observations of herbarium specimens at AA, ALTB, BM, E, IRK, K, LE, MHA, MW, NENU, NS, NSK, TK, UUH, VBGI, and VLA, including the original material for the names studied; and from the relevant species descriptions available in the literature [32,45,95,96,97]. The rhizome and root diameter were measured in the dry state with a digital Vernier caliper Series 532 (Mitutoyo, Aurora, IL, USA). Because *I. psammocola* is not represented in the Chinese botany databases [54,98,99], its taxonomy is based on a comprehensive study of the protologue.

### 2.5. Taxonomy and Distribution

The conservative taxonomy of *Iris* was used [5,6,7,8,10,21,30,35,39,45,50,66,67,96]. For the nomenclature, the relevant articles and recommendations of the *Shenzhen Code* [100] were consulted. We used the name *I. potaninii* var. *ionantha*, as its taxonomy is controversial and needs further research (see Introduction).

In the Taxonomic Treatment section (see below), we gathered information on the distribution of the accepted species from our own field data, the herbarium specimens, and relevant literature [47] and critically assessed the collection points a priori from social networks [98,99,101,102,103].

## 3. Results

### 3.1. Genetic Divergence and Phylogenetic Relationships within Iris Sect. Psammiris

Four cpDNA regions were sequenced from 72 accessions of 10 *I*. sect. *Psammiris* species, 4 accessions of *I. tigridia*, 7 accessions of *I. ivanovae*, 2 accessions of *I. goniocarpa*, and 1 accession of *I. potaninii* var. *ionantha*, as well as from 2 samples of unknown species. A total of 18 haplotypes were identified among the samples from 10 species of *I*. sect. *Psammiris*, *I. tigridia*, and *I. ivanovae* based on polymorphic sites found at 3783 aligned positions of a combined dataset. The distribution of these haplotypes among the studied species is shown in Figure 2.

A total of 6 haplotypes (H1–H4, H6, and H7) were identified in 25 localities of *I. potaninii*, 3 haplotypes occurred in 17 localities of *I. humilis* (H9–H11), and 3 haplotypes occurred in 3 localities of *I. kamelinii* (H1, H3, and H8). Two haplotypes were found in *I. psammocola* (H1 and H5), *I. arenaria* (H12 and H13), *I. schmakovii* (H9 and H11), and *I. ivanovae* (H17 and H18); the following species showed one haplotype each: *I. pineticola* (H9), *I. bloudowii* (H15), *I. mandshurica* (H14), *I. vorobievii* (H16), and *I. tigridia* (H17). Of the six haplotypes found in *I. potaninii*, haplotype H7 was shared by the accessions from ZTLW, ZTLN, and ZAC; haplotypes H2 and H4 were shared by the accessions from two localities; and the accessions from the other 16 localities of *I. potaninii* shared a single common haplotype, i.e., H1. The latter was found to be common to three species: *I. potaninii*, *I. psammocola*, and *I. kamelinii*. Another haplotype (H3) was common to *I. potaninii* and *I. kamelinii* (localities MKS and MAK, respectively). Of the three haplotypes found in *I. humilis*, haplotype H9 proved to be the most frequent: it was shared by the accessions from 11 out of 17 localities. Moreover, this haplotype was also found in both studied localities of *I. pineticola*, while *I. schmakovii* shared two haplotypes (H9 and H11) with *I. humilis*.

**Figure 4:**
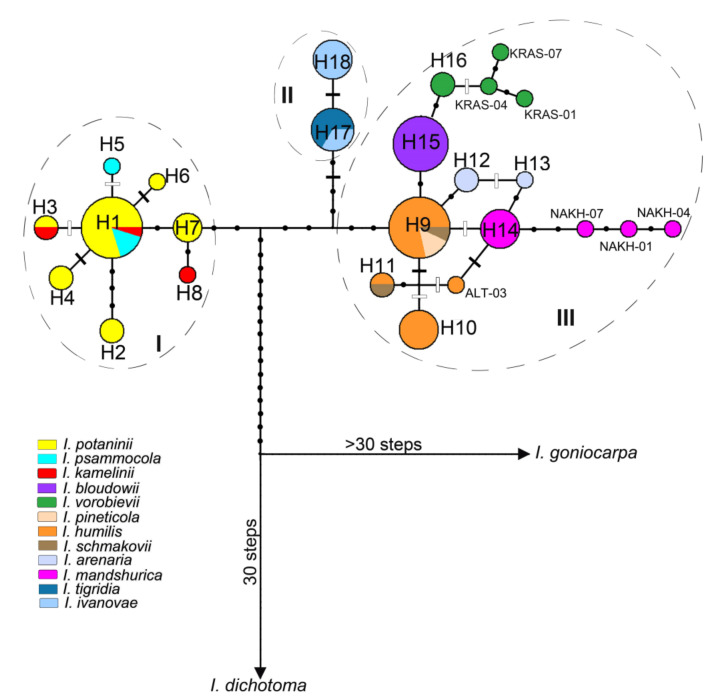
Median-joining network inferred from combined sequences of the *trnH-psbA*, *rps4-trnS*^GGA^, *trnS-trnG*, and *trnL-trnF* regions showing the relationships among the cpDNA haplotypes of the *Iris* sect. *Psammiris* species, *I. tigridia*, *I. ivanovae*, and haplotypes of *I. goniocarpa* with *I. dichotoma* as outgroups. Each circle indicates a haplotype, with the size of the circle proportional to the number of localities where this haplotype was found. Black dots indicate nucleotide substitutions; thick white and black bars depict 1 bp and multi-base indels, respectively; the haplotypes outlined by dashed lines shows haplogroups I–III within *I.* sect. *Psammiris*. For haplotype codes, see Table 1.

The genealogical relationships between the haplotypes of the studied species are shown in Figure 4. All the haplotypes, including NAKH-01, NAKH-04, NAKH-07, KRAS-01, KRAS-04, KRAS-07, and ALT-03 retrieved from reference [34] and the haplotypes of *I. goniocarpa* and *I. dichotoma*, were connected in a single network. All of them, except for the haplotypes of *I. goniocarpa*, were closely related and originated from the same unsampled or extinct ancestral haplotype connected via many mutational steps with the haplotype of *I. dichotoma*. Three haplogroups were detected in the network, separated from each other by several mutational steps (six to eight). Haplogroup I included closely related haplotypes H1–H8 arranged into a star-like pattern around haplotype H1, which was common to *I. potaninii*, *I. kamelinii*, and *I. psammocola*. The pairwise *F*_ST_ values between these species were not significant (*p* > 0.1), no nucleotide substitutions or indels differentiating these species were revealed, and *K*_S_ between them varied from 0.00006 to 0.00015, indicating a lack of genetic differences between these species.

The other two haplogroups (II and III) descended from a haplotype that may be either extinct or missing from the current sampling. The pairwise *F*_ST_ value between these groups was 0.782 (*p* = 0.00001), and the *K*_S_ between them was 0.00126. Four nucleotide substitutions and 8 bp insertion distinguished the species from these groups. Haplogroup II included two haplotypes found in *I. tigridia* and *I. ivanovae*, of which one was common (H17) and the other (H18) was found only in samples from the *I. ivanovae* localities, differing from H17 by an insertion of 25 bp within the *trnH-psbA* spacer. The low and non-significant *F*_ST_ value (*p* > 0.05) between *I. tigridia* and *I. ivanovae* and the absence of sequence divergence between them (*K*_S_ = 0.0000) may indicate that they belong to the same species.

Haplogroup III included 14 closely related haplotypes found in 7 species: *I. arenaria*, *I. bloudowii*, *I. humilis*, *I. mandshurica*, *I. pineticola*, *I. schmakovii*, and *I. vorobievii*. Haplotypes in this haplogroup differed from the neighboring haplotypes by one or two mutational steps. The most frequently occurring haplotype (H9) occupied a central position in this haplogroup and was common to most *I. humilis* accessions from different parts of the range, as well as to the accessions from the two *I. pineticola* localities and to one *I. schmakovii* accession (Figure 2 and Figure 4). Many haplotypes of haplogroup III were connected with H9 via one (H12 of *I. arenaria*, H14 of *I. mandshurica*, and H15 of *I*. *bloudowii*) or two mutational steps (H10, H11, and ALT-03 of *I. humilis*), forming a star-like structure. The haplotypes of *I. mandshurica*, which were interconnected via one or two mutational steps, were also closely related to the haplotypes of *I. humilis.* Alternative connections (loops in the network) between some haplotypes, including the most common haplotype (H9), indicated a homoplasy that hampered unambiguous identification of genetic relationships between the haplotypes of *I. mandshurica* and *I. humilis*. The haplotypes of *I. vorobievii* formed a group with a single haplotype of *I. bloudowii* (H15), which differed from the most common haplotype (H9) by a single substitution.

**Figure 5:**
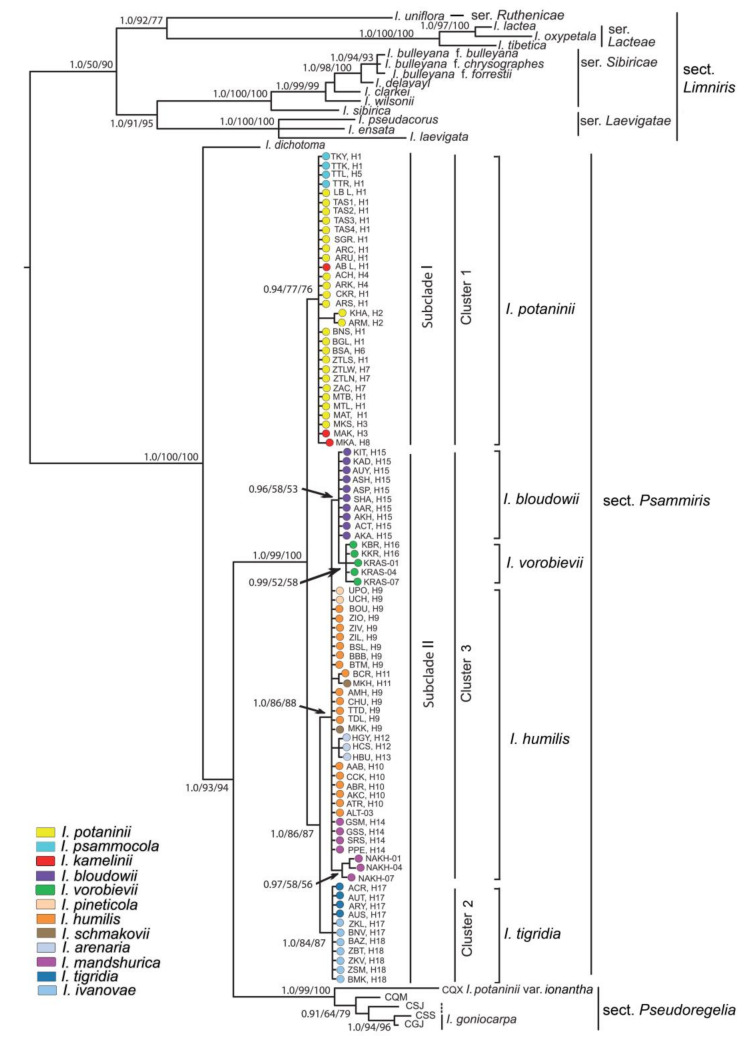
The Bayesian majority rule consensus tree of the *Iris* sect. *Psammiris* samples inferred from combined *trnH-psbA*, *rps4-trnS*^GGA^, *trnS-trnG*, and *trnL-trnF* chloroplast data. The numerals above the branches are Bayesian posterior probabilities (PP > 0.9) and bootstrap values (>50%) for the MP and ML methods. The haplotype and locality codes correspond to those listed in Table 1.

Trees with nearly identical topologies and with slight differences in statistical supports of some nodes were inferred by the MP, ML, and BI methods based on the cpDNA dataset (Figure 5). In these trees, all the accessions were distributed with a robust support (PP 1.0, BP > 90%) in accordance with their affiliation to the corresponding sections of the genus *Iris*. The sister-group relationship between *I.* sect. *Pseudoregelia* and *I*. sect. *Psammiris* was strongly supported (PP 1.00, BP 93, and 94%). The position of the CQM and CSJ accessions in the *I.* sect. *Pseudoregelia* group, together with *I. goniocarpa* and *I. potaninii* var. *ionantha*, was also strongly supported (PP 1.00, BP 99, and 100%).

The species of *I*. sect. *Psammiris* formed a monophyletic clade (PP 1.00, BP 99, and 100%), with *I*. *tigridia* and *I. ivanovae* nested within it. This clade was divided into two sister subclades, with the nucleotide divergence (*K*_S_) between them being 0.00182. Subclade I corresponded to haplogroup I revealed by the MJ methods (Figure 4) and included all the samples of *I. potaninii*, *I. psammocola*, and *I. kamelinii*, with moderate support (PP 0.94, BP 77, and 76%). Subclade II combined *I. tigridia*, *I. ivanovae*, and all other species recognized in *I*. sect. *Psammiris* (PP 1.00, BP 86, and 87%). This subclade, in turn, was divided into two well-supported clusters, of which one (cluster 2, support values of PP 1.0, BP 84, and 87%) contained the samples of *I. tigridia* and *I. ivanovae*, while the other (cluster 3, PP 1.0, BP 86, and 88%) included haplotypes of seven species (*I. arenaria*, *I. bloudowii*, *I. humilis*, *I. mandshurica*, *I. pineticola*, *I. schmakovii*, and *I. vorobievii*). These two clusters corresponded to haplogroups II and III revealed by the MJ methods. A low nucleotide divergence was observed between the species within cluster 3 (*K*_S_ ranged from 0.00001 to 0.00088), and the relationships between *I. arenaria*, *I. humilis*, *I. mandshurica*, *I. pineticola*, and *I. schmakovii* remained unresolved. Only the haplotypes of *I. bloudowii* and *I. vorobievii* formed a group that received weak support from the MP and ML methods (BP 58, 53%) and high support only from the BI method (PP 0.96).

Thus, the results of this study based on sequencing of the cpDNA regions of 12 taxa show that *I*. sect. *Psammiris* includes five species (*I. bloudowii*, *I. humilis*, *I. potaninii*, *I. tigridia*, and *I. vorobievii*) and is divided into three groups.

### 3.2. Morphological Comparison of the Iris Sect. Psammiris Species

A detailed morphological comparison among the *I.* sect. *Psammiris* species accepted in the present study is listed in Table 2 (also see Table S1). They can be easily distinguished by the number of flowers and bracteoles, as well as by the stem height and perianth tube length.

**Figure 6:**
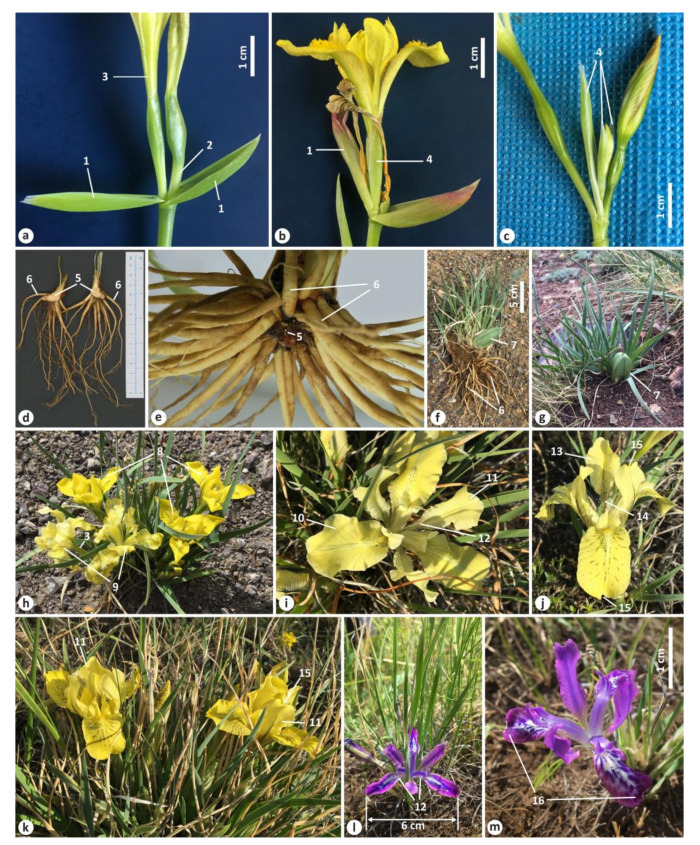
Morphological features used for characterization of *Iris* sect. *Psammiris*: (**a**–**c**) *I. humilis*, inflorescence structure ((**a**,**b**) Russia, Zabaykalsky Krai, vicinities of the Orlyonok flag station; (**c**) Russia, Republic of Buryatia, Mount Spyashchiy Lev [bracts were removed]); (**d**,**e**) *I. vorobievii*, underground organs (Russia, Primorsky Krai, vicinities of Kraskino); (**f**,**g**) *I. potaninii*, in fruiting (Russia, Altai Republic, vicinities of Chagan-Uzun); (**h**–**k**) *I. potaninii*, flower morphology ((**h**) Russia, Republic of Buryatia, vicinities of Novoselenginsk; (**i**,**j**) Russia, Republic of Buryatia, vicinities of Gusinoye Lake; (**k**) (= *I. kamelinii*) Russia, Altai Republic, vicinities of Verkhniye Boguty Lake); (**l**,**m**) *I. tigridia* (= *I. ivanovae*), flowers (Russia, Zabaykalsky Krai, vicinities of Kharanor). Marks are as follows: 1, bract; 2, pedicel; 3, perianth tube; 4, bracteole; 5, rhizome; 6, adventitious root; 7, fruit; 8, bright yellow flower; 9, pale yellow flower; 10, blade of fall; 11, obovate standard; 12, gradually narrowing claw; 13, elliptic standard; 14, abruptly narrowing claw; 15, notch; 16, underdeveloped blade of fall. Photos by E. Boltenkov.

*Iris bloudowii*, *I. humilis*, and *I. vorobievii*, forming a group of related species, are distinguished as having non-contractile, adventitious roots; thin and broad rosette leaves, usually with more than one flower and with bracteoles; tough green bracts; long pedicels; and a short perianth tube. Among them, *I. humilis* is similar to *I. bloudowii* but differs in its habit (less robust), number of bracteoles (from their absence to three; Figure 6a–c), and number of flowers (up to three; Figure 6b,c); moreover, it occasionally occurs in clumps (Figure 1c). In addition, a white-flowered form of *I. humilis*, a rare feature in this section, was found in Partizansky (S. Prokopenko, pers. comm.) and Khankaysky (A. Malyk, pers. comm.; Figure 1b) districts, Primorsky Krai, Russia. *Iris vorobievii* is characterized by a very short rhizome (up to 2 cm in length), storage-like roots spreading almost horizontally (Figure 6d,e), and often branched stems (e.g., VBGI79851; see http://botsad.ru/herbarium/, accessed on 20 December 2022).

*Iris potaninii* is similar to *I. tigridia* in the characters of roots (contractile), rosette leaves (ensiform, acute, or narrowly acute at the apex; tough; narrow; 0.1–0.6 cm wide), stem (with non-curled remains of leaves at the base, simple, 1-flowered, without bracteoles), bracts (lanceolate, whitish, and thin), pedicel (extremely short, less than 0.7 cm), and elliptical fruit. However, *I. potaninii* is distinguished from *I. tigridia* by its shortened, branching rhizomes; by having fibrous remains of leaves (vs. the rhizome surface glabrous); often forming large colonies or clumps (Figure 1g,h); by longer (up to 50 cm long), slightly less thickened roots; by a much shorter stem (to 2.5 cm long) not emerging above ground, resulting in fruit always being borne at the soil surface (Figure 6f,g); by having an extremely short internode between the upper cauline leaf and bracts (barely 0.2 cm long); and by its even longer perianth tube (usually more than two times as long as that of other species in the section). After opening, the color of *I. potaninii* flowers is bright yellow, subsequently turning into pale yellow (Figure 1g and Figure 6h, respectively). The species is variable in the color intensity of broken lines (brownish against yellow background) of the fall blade (Figure 6i–k), the shape (obovate or elliptic) of the inner perianth segments (or standards), in terms of whether they gradually or abruptly narrow into the claw (Figure 6i,j), and in terms of whether falls and standards have a notch at the apex (emarginated) (Figure 6i–k). Variability in these characters can be observed within the same locality or even clump.

*Iris tigridia* is clearly distinguished by its flower color, varying from pale blue to dark blue and purple or, rarely, white (Figure 1i–m and Figure 6l,m); however, it is never yellow as others in *I.* sect. *Psammiris*. Generally, it is variable (within a locality) in leaf length and width, stem height, and cauline leaf length (Table 2), as well as in floral diameter (3.5–6 cm).

## 4. Discussion

This study presents the most comprehensive phylogenetic analysis for *I.* sect. *Psammiris* of those carried out to date. The reported results provide new insights into the taxonomic composition and classification of this section. The samples represent almost all known taxa (a total of 12) in *I.* sect. *Psammiris*. Specimens of the species closely related to *I. humilis* (*I. arenaria*, *I. pineticola*, and *I. schmakovii*) and *I. potaninii* (*I. kamelinii* and *I. psammocola*) were included in the phylogenetic analysis for the first time. Our sampling of all the currently recognized species of *I.* sect. *Psammiris* from different parts of the ranges and type localities made it possible to clarify the genetic relationships between them, as well as with *I. tigridia* and *I. ivanovae*, which are now considered as representatives of *I.* sect. *Pseudoregelia* [1,11,67,68].

The monophyly *I.* sect. *Psammiris* has been questioned by other authors [14,50,51,66], since *I. potaninii* var. *ionantha* was shown to be related to *I*. sect. *Pseudoregelia*. The findings of this study clearly show that all the specimens of *I. tigridia* and its close relative, *I. ivanovae*, belong to the clade of *I.* sect. *Psammiris* (Figure 4 and Figure 5), which is monophyletic and sister to the clade formed by the taxa of *I*. sect. *Pseudoregelia*, including *I. potaninii* var. *ionantha*. The phylogenetic placement of *I. potaninii* var. *ionantha* is fully congruent with the tree topologies inferred in recent phylogenetic studies [14,50,51].

Within the *I*. sect. *Psammiris* clade, we revealed three well-supported monophyletic groups (haplogroups in the MJ network and clusters in the phylogenetic tree) treated by us at the series level, two of which we consider unispecific. The first group includes *I. kamelinii*, *I. potaninii*, and *I. psammocola*. These species have common or closely related haplotypes and demonstrate the lack of clear differentiation from each other (Figure 4 and Figure 5). Therefore, the first group can be considered unispecific. We choose *I.* sect. *Psammiris* ser. *Potaninia* Doronkin to represent the group, as it has the same type (*I. potaninii*) as Doronkin’s original group [11]. A thorough revision of the morphological characters previously proposed to distinguish between these species confirmed the lack of clear differences.

To date, eight species have been recognized in *I*. sect. *Psammiris* [15,17]. However, the taxonomic statuses of *I. kamelinii*, *I. psammocola*, *I. arenaria*, *I. mandshurica*, *I. pineticola*, and *I. schmakovii*, as well as *I. ivanovae*, are considered controversial.

The following diagnostic features were used to distinguish *I. kamelinii* from *I. potaninii*: rhizomes bear membranous remains of leaf bases (vs. fibrous remains); standards rounded–elliptic, emarginated at apex, abruptly narrowed into a linear claw (vs. obovate, gradually narrowed into a claw); and ornamentation of falls with a dense pattern of purple veins (vs. veins poorly visible) [61]. In the present study, we clearly showed that these features of *I. kamelinii* are identical or slightly differ from those of *I. potaninii* (see Section 3.2); thus, *I. kamelinii* does not have any diagnostic features that clearly distinguish it as a distinct species. It has long been noted that the standards in *I. potaninii* are usually emarginated at the apex [95,96,97]. Our data confirm that the standards in *I. potaninii* can be emarginated (or not) at the apex, even within the same plant from the type locality of *I. kamelinii* (Figure 6k); fall ornamentation is a variable character in *I. potaninii* (Figure 6i–k). As a consequence, we regard *I. kamelinii* as a synonym of *I. potaninii*.

**Figure 7:**
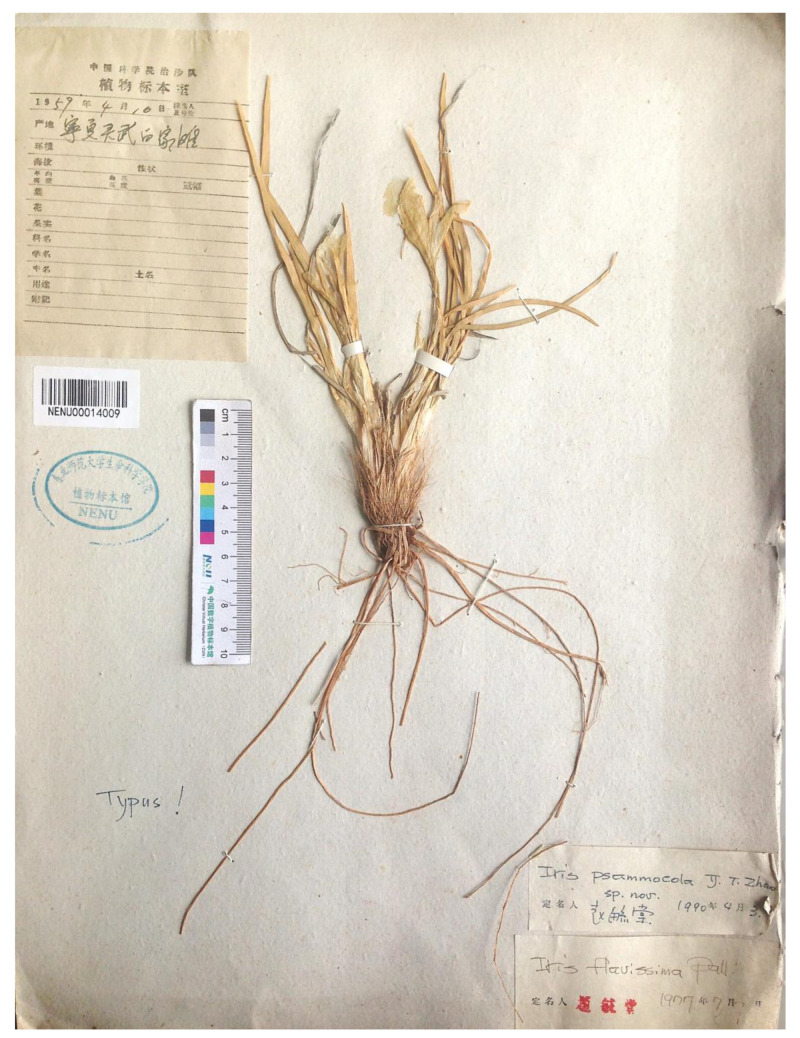
Holotype of *Iris psammocola* (NENU00014009) (included with permission of the curator).

The plants of *I. psammocola* from the Republic of Tuva, Russia [55], and the plants from the type locality of *I. kamelinii* [61] are found growing together with *I. potaninii* and all have the same chromosome number, i.e., 2*n* = 22 (see Introduction). In China (where *I. psammocola* was described), this species is known to date only from the protologue consisting of a diagnosis, a description, and an illustration [53]; from a single specimen deposited at NENU (NENU00014009!; Figure 7), which is a holotype of the name; and from reference [46]. The holotype of *I. psammocola* is represented by a small herb plant in flowering collected in early April. This specimen has a rhizome (broken) of about 0.5 cm in diameter; its adventitious roots are yellowish white, thickened at the proximal part, and gradually tapering to the apex, up to 20 cm long; the rosette leaves are ensiform, narrowly acute at the apex, tough, and finely ribbed, up to 18.5 cm long and 0.2–0.4 cm wide; the flowering stem is very short, not emerging above ground, probably not more than 2 cm tall (the height was impossible to measure), simple, bearing one terminal flower, and without bracteole; the stem and rhizome bear erect (non-curled) fibrous remains of leaves; two bracts are lanceolate and membranous; the pedicel is very short; the perianth tube is filiform, about 5 cm long; the outer perianth segments have a distinct beard, about 4.5 cm long. According to references [46,53], the rhizome of *I. psammocola* is short and non-stoloniferous, the bracts are 3.5–4 cm in length, and the flowers are yellow. After a critical examination of the *I. psammocola* protologue, we found that the features of the rhizome, roots, rosette leaves, flowering stem, bracts, and flowers are identical to those of *I. potaninii* (Table 2). Our analysis of cpDNA variability indicate the lack of genetic differences between the specimens of *I. potaninii* and the specimens from the Republic of Tuva, Russia, including the specimen (TTL) treated as *I. psammocola* (Figure 4 and Figure 5).

The phylogenetic analysis reported above (Figure 4 and Figure 5) confirmed that the plants from the type locality of *I. tigridia* and the plants from the Republic of Buryatia and Zabaykalsky Krai, here named as *I. ivanovae*, belong to the same species, *I. tigridia*, which is nested in *I.* sect. *Psammiris* and comprises a separate unispecific series. To describe *I. ivanovae* based on plants from Zabaykalsky Krai, Russia, the following diagnostic features were used to distinguish it from *I. tigridia*: flowers 2.5–3.5 cm in diameter (vs. flowers 4.0–6.0 cm in diameter); falls abruptly narrowed into a long, filiform claw (vs. falls gradually narrowed into a thin claw); bracts narrowly lanceolate, gradually acuminate (vs. bracts oblong-elliptical and short-pointed at the apex); and leaves gradually acuminate, 0.1–0.2 cm wide (vs. leaves shortly acuminate and 0.4–0.5 cm wide) [73]. However, our field study at the type locality of *I. ivanovae* did not confirm some of these features. We found that the flowers were mainly 3.5–6.0 cm in diameter, the falls were gradually (not abruptly) narrowed into a thin claw, 0.1 cm wide at the base, and the leaves were 0.1–0.3 cm wide (Figure 6l). The diameter of one of the ten flowers that we found was only 2.5 cm due to the underdeveloped blades of falls at the apex (Figure 6m). This plant can be considered merely an aberrant, which can be explained by the climatic conditions of the locality where it grew. In addition, we found that the lanceolate, gradually acuminate bracts are characteristic of all the plants of *I. tigridia* from Siberia, as well as the plants with leaves gradually narrowed to the apex. However, to the best of our knowledge, the latter dominate the Transbaikalian steppes and Mongolian habitats due to the rather xerophytic conditions. Furthermore, leaves in *I. tigridia* were previously characterized as gradually narrowed to the apex [45,95,96]. Thus, we did not find any differences between the plants from the type locality of *I. ivanovae* and the plants from the *I. tigridia* distribution range, which confirmed Gubanov’s opinion [76] that *I. ivanovae* is a synonym of *I. tigridia*.

The third group, revealed by the MJ methods and phylogenetic analyses of *I.* sect. *Psammiris*, comprises seven species: *I. arenaria*, *I. bloudowii*, *I. humilis*, *I. mandshurica*, *I. pineticola*, *I. schmakovii*, and *I. vorobievii* (Figure 4 and Figure 5). The isolated position of *I. bloudowii* and *I. vorobievii* in this group, along with the data on their morphology presented here (Table 2), are consistent with the results of previous studies [15,27,34,46,47,48] that showed them as separate species. Phylogenetic relationships between *I. arenaria*, *I. humilis*, *I. mandshurica*, *I. pineticola*, and *I. schmakovii* from Hungary, Ukraine, Mongolia, and Russia (Belgorod Oblast, Altai Krai, Altai Republic, Republic of Tuva, Republic of Buryatia, Zabaykalsky Krai, Amur Oblast, and Primorsky Krai) remain unresolved, and our results (the shared haplotypes and the star-like pattern) indicate a lack of clear genetic differentiation between them, which suggests that they belong to a single species, *I. humilis*.

The taxonomy of *I. humilis* has long been debated and based exclusively on traditional morphological study. An opinion existed that, having an extensive distribution range, *I. humilis* could not be homogenous; therefore, its numerous varieties were not considered as separate species [10,12,22,23]. Our data agree with the suggestion expressed by many authors on the taxonomy of *I. arenaria* [10,13,22,23,24,26,35], *I. mandshurica* [32,33], and *I. pineticola* [1,10,12,13,26,40] as taxonomic synonyms of *I. humilis*.

*Iris humilis* var. *umbrosa* was described based on plants collected on the right bank of Lake Khuvsgul, Khuvsgul Aimag, Mongolia. As follows from the brief description, it is a plant with a height of 20–30 cm; green linear–lanceolate leaves 3–8 mm wide ; yellow flowers with purple veins ; bracts coriaceus ; wide, swollen, acuminate, and fruit elliptical, tapering at the apex [70]. Unfortunately, the taxon *I. humilis* var. *umbrosa* was published without a diagnosis, and in our opinion, it is still unclear what distinguishes it from the autonymic variety. Moreover, all the features indicated in the protologue of *I. humilis* var. *umbrosa* and in reference [17] are identical to those of *I. humilis* (Table 2). All experts on the Mongolian flora listed *I. humilis* for the Khuvsgul phytogeographical region [27,74,75,76,77]. Despite the considerations mentioned above, Alexeeva referred to a “more detailed comparative morphological analysis of characters” (that she, however, never presented) and came to the conclusion that *I. humilis* var. *umbrosa* is actually a new species, *I. schmakovii* [17]. In accordance with the molecular data presented here, *I. schmakovii* belongs to *I. humilis* (Figure 4 and Figure 5).

It should also be noted that four cytotypes are known for *I. humilis*: 2*n* = 22, 24, 26, and 28. The following geographic pattern of the distribution of these cytotypes can be observed. For instance, the cytotype 2*n* = 22 has been recorded from the European part of the distribution range, e.g., from Ukraine (sub *I. pineticola*) [59] and Czech Republic (sub *I. arenaria*) [37]; 2*n* = 28 has been reported primarily from the central part of the distribution range, e.g., from Mongolia (sub *I. flavissima*) [104] as well as from Tomsk Oblast [105], Altai Republic [58], Republic of Tuva [60], Irkutsk Oblast [106], and Republic of Buryatia, Russia [45]; 2*n* = 24 has been reported from the eastern part of the distribution range, e.g., from the Republic of Buryatia [107] and Primorsky Krai (sub *I. mandshurica*) [108]. The cytotype 2*n* = 26 has been reported for Altai Republic, Russia (sub *I. bloudowii*) [57], and Jilin Province, China (sub *I. bloudowii*) [109]. In addition, two cytotypes, i.e., 2*n* = 24 and 2*n* = 28, have been recorded from plants collected at the same localities of Primorsky Krai, (sub *I. mandshurica*) [110,111] and Amur Oblast, Russia [58,112].

### 4.1. Taxonomic Treatment

In the present study, we propose *I*. sect. *Psammiris* to be divided into three series consisting of five species. In particular, we confirm that *I. tigridia*, the type species of *I*. sect. *Pseudoregelia* ser. *Tigridiae* [11], is nested in *I*. sect. *Psammiris*. Therefore, we suggest excluding *I*. ser. *Tigridiae* from *I*. sect. *Pseudoregelia*, as originally published in [11], and transferring it to *I*. sect. *Psammiris*. In addition, *I*. ser. *Humiles* Doronkin and *I*. ser. *Vorobievia* Alexeeva are synonymized here for the first time with the autonymic series of *I*. sect. *Psammiris*.

Moreover, as found in the present study, there are some problems related to the type citation in *I*. sect. *Psammiris*; therefore, the following issues should be addressed:

(i) Taylor indicated *I. humilis* as the type species of *I*. sect. *Psammiris* [9]. Since then, this approach has been accepted [11,12,14,15,71,113]. However, *I.* subgen. *Psammiris* was actually published by Spach as a monotypic taxon based on *I. arenaria*, although he noted this group to apparently also include *I. flavissima* and *I. bloudowii* as follows: “Huc referendae etiam videntur *Iris flavissima*, Jacq., et *Iris Bloudowii*, Ledeb.” [3]. We tend to interpret this phrase as non-inclusion of *I. flavissima* and *I. bloudowii* in *I.* subgen. *Psammiris* by Spach. He did not include these species in *I.* subgen. *Psammiris* either in the following study published four months later [114], which can be considered an indirect argument in favor of our opinion. The type of *I. arenaria*, not *I. humilis*, is therefore, the type of *I*. sect. *Psammiris* (see Art. 10.3 of the ICN).

(ii) The herbarium sheet at MW (MW0021793!) with a label handwritten by Georgi (“*Iris pumila* ad Baical, 1772”), which is the current lectotype of *I. humilis* [115], consists of four plants representing two species: *I. pumila* L. and *I. humilis* (as currently applied). When a type contains parts belonging to more than one taxon, the initial choice is superseded (see Art. 9.19 of the ICN), and the name must remain attached to the part that corresponds most nearly with the original description or diagnosis (Art.  9.14 of the ICN). Hence, because MW0021793 proved to be mixed and belong to more than one taxon, it cannot be accepted as a type of *I. humilis*, as previously proposed [115]. One of us (E.V. Boltenkov) numbered the plants belonging to *I. humilis* from the Lake Baikal area as 1 and 2, which was noted by Alexeeva [15], and the plants belonging to *I. pumila* of unknown origin as 3 and 4. However, Alexeeva [15] did not achieve the type designation because the typification statement did not include the phrase “designated here” or an equivalent (see Art. 7.11 of the ICN).

(iii) Contrary to the Alexeeva’s statements [116], the type of *I. tigridia* was not indicated by Grubov [27] (see Art. 40 Note 2). Similarly, the type designation of this name was not effectively published by Alexeeva [15,116], as required by Art. 7.11 of the ICN.

(iv) *Iris pineticola* was published [39] (p. 407) as a replacement name for *I. flavissima* subsp. *stolonifera* f. *orientalis* Ugr. Hence, the latter name is its replaced synonym (Art. 6.11 of the ICN) that has the same type as that of the replacement name (see Art. 7.4 of the ICN). Klokov indicated the specimen deposited at KW as the “typus speciei” of *I. pineticola* as follows: “RSS Ucr., dit. Charcoviensis, in pineto prope pag. Choroshevo, 5–6 V. 1855. Legit B.M. Czernjajev; in Herbario Instituti Botanici Ac. Sc. RSS Ucr. conservatur” [39]. While preparing his publication [22], Ugrinsky used the Vassilii Czernajew’s herbarium; therefore, Klokov’s indication could have been accepted as the lectotype for *I. flavissima* subsp. *stolonifera* f. *orientalis*, satisfying the requirements of Art. 7.11 of the ICN. However, Czernajew’s specimen cited by Klokov [39] was lost or destroyed, and for this reason, a neotype of *I. pineticola* (KW000114271) was selected (see Art. 9.16 of the ICN) [41]. Unfortunately, the authors of the latter paper did not consider all the original material in the context of the protologue of *I. flavissima* subsp. *stolonifera* f. *orientalis* (see Arts. 9.4 and 9.13 of the ICN), which contains an illustration [22] (p. 307). The same illustration was provided by Klokov in reference [39] (p. 293). In accordance with Art. 9.19 of the ICN, the choice of the neotype [41] should be superseded since the original material (illustration) was found to exist and can serve as lectotype.

As a consequence, lectotypes are designated here for *I. flavissima* subsp. *stolonifera* f. *orientalis*, *I. humilis*, and *I. tigridia*.

#### 4.1.1. List of Taxa

Below is a list of the accepted species (highlighted in bold italics) that contains information on their synonyms and nomenclatural types, as well as on their distributions, habitats, and chromosome numbers.

***Iris*** sect. ***Psammiris*** (Spach) J.J. Taylor, Proc. Biol. Soc. Washington 89(35): 417, 1976 ≡ *I.* subgen. *Psammiris* Spach, Ann. Sci. Nat., Bot., ser. 3, 5(1): 110, 1846.—Type species: *Iris arenaria* Waldst. et Kit.

(I) Iris ser. Psammiris

= *Iris* ser. *Humiles* Doronkin, Bot. Zhurn. 75(3): 415, 1990, ***syn. nov***.—Type species: *Iris humilis* Georgi.

= *Iris* ser. *Vorobievia* Alexeeva, Phytotaxa 340(3): 205, 2018, ***syn. nov***.—Type species: *Iris vorobievii* N.S. Pavlova.

(1) ***Iris humilis*** Georgi, Bemerk. Reise Russ. Reich 2: 196, 1775.—Lectotype (designated here by E.V. Boltenkov): [Russia, Irkutsk Oblast] ad Baikal, [fl.], 1772, [*Georgi*] *s.n*. Herb. C.B. Trinius (MW0021793!, sub “*Iris pumila* L.” det. J.G. Georgi).—https://plant.depo.msu.ru/open/public/en/item/MW0021793 (accessed on 20 December 2022).

= *Iris flavissima* Pall., Reise Russ. Reich. 3(2): 715, 1776.—Lectotype (designated by Alexeeva [115] (p. 917)): [Russia, Zabaykalsky Krai] *Iris* lutea biflorae affinis, Dahuria, [fl.], [June 1772], [*Pallas*] *s.n*. Herb. P.S. Pallas (BM000832584!).—https://data.nhm.ac.uk/dataset/collection-specimens/resource/05ff2255-c38a-40c9-b657-4ccb55ab2feb?q=BM000832584 (accessed on 20 December 2022).

= *Iris mandshurica* Maxim., Bull. Acad. Imp. Sci. Saint-Pétersbourg 26(3): 530, 1880.—Lectotype (designated by Alexeeva [116] (p. 417)): [Russia, Primorsky Krai], [handwritten by Goldenstädt]: In der Nähe von Nikolske, auf Sandboden, gelb, [fl.], 14 May 1872, [*Goldenstädt*] *19*; [handwritten by C.J. Maximowicz]: *Iris mandshurica* Maxim. Suifun, Mandshuriae, *Goldenstädt* (LE01025688! cum icon, isolectotype LE01010784!).—http://re.herbariumle.ru/01025688 (accessed on 20 December 2022).

= *Iris arenaria* Waldst. et Kit., Descr. Icon. Pl. Hung. 1: 57, 1802 ≡ *I. humilis* subsp. *arenaria* (Waldst. et Kit.) Á.Löve et D.Löve, Bot. Not. 114(1): 51, 1961.—Lectotype (designated by Alexeeva [15] (p. 207)): [illustration] “*Iris arenaria*” in Waldstein et Kitaibel [117] (t. 57).—https://bibdigital.rjb.csic.es/records/item/11187-redirection (accessed on 20 December 2022).

= *Iris flavissima* subsp. *stolonifera* f. *orientalis* Ugr., Trudy Obsc. Isp. Prir. Imp. Har’kovsk. Univ. 44: 305, 1911 ≡ *I. pineticola* Klokov, Fl. URSR 3: 407, 1950.—Lectotype (designated here by E.V. Boltenkov): [illustration] “*Iris flavissima* Pall. I. B. *orientalis* Ugr.” in Ougrinsky [22] (p. 307).—https://www.biodiversitylibrary.org/item/26298#page/319/mode/1up (accessed on 20 December 2022).

= *Iris humilis* var. *umbrosa* Alexeeva, Turczaninowia 14(1): 59, 2011 ≡ *I. schmakovii* Alexeeva, Turczaninowia 21(4): 145, 2018, ***syn. nov***.—Holotype: Mongolia, Khuvsgul Aimag, the right bank of the Khuvsgul Lake, 50°34′ N 100°28ʹ E, 1,738 m, [fr.], 6 July 2007, *R.V. Kamelin* et al. *23* [originally in Russian] (LE01042608!).—http://re.herbariumle.ru/01042608 (accessed on 20 December 2022).

Distribution and habitat: *Iris humilis* is the most widely distributed and northernmost of *I*. subgen. *Iris* and is the only arillate iris native to Europe. Its range stretches along the Eurasian steppe belt from Europe to the Pacific coast, including the steppe patches of southern Siberia and the Russian Far East. It is found from Central and Eastern Europe (northeastern Austria, southern Czech Republic, Hungary, northern Romania, Slovakia, and Ukraine), including the Central Black Earth Economic Region and eastern oblasts of the Volga region and Pre-Urals, to southern Siberia, Russia, and northern Kazakhstan, as well as in northern Mongolia, northeastern China (northeastern Inner Mongolia and Heilongjiang, Jilin, and Liaoning provinces), North Korea (Ryanggang, Jagang, and Kangwon provinces), southern Russian Far East, and eastwards to the Pacific coast, where it has been recorded from dunes near the Kievka River estuary (Primorsky Krai, Russia). The northernmost wild locality of *I. humilis* has been found in vicinities of Kochegarovo Village (Olekminskiy District, Yakutia, Russia, at approximately latitude 60° N; N.S. Danilova, pers. comm.). *Iris humilis* is characterized by good adaptation to sandy, stony, clayey, limestone, and humus-rich soils. It grows commonly in open places in steppes and meadows, on slopes, at edges of pine forests, and along river banks at elevations of 350–1850 m.

Chromosome numbers: 2*n* = 22, 24, 26, and 28 (see below).

(2) ***Iris bloudowii*** Ledeb., Icon. Pl. [Ledebour] 2: 5, 1830 ≡ *I. flavissima* α [var.] *umbrosa* Bunge, Fl. Altaic. [Ledebour] 1: 60, 1829, excl. syn. ≡ *I. flavissima* var. *bloudowii* (Ledeb.) Baker, Handb. Irid.: 29, 1892.—Lectotype (designated by Sennikov et al. [113] (p. 31)): [Kazakhstan, East Kazakhstan Region] ad Grammahuham [Gromotukha River], [fl.], 4 May 1826, [*Ledebour*] *95*, Herb. C.F. Ledebour (LE01010770!, sub “*Iris* [*flavissima*, originally] *bloudowii* m.” det. Ledebour).—http://re.herbariumle.ru/01010770 (accessed on 20 December 2022).

Distribution and habitat: This species is found in the Altai-Sayan region, Northern Tian Shan (Dzhungraian Alatau and Kungey Alatau) and Inner Tian Shan (Terskey Ala-Too) and is distributed in northern Kyrgyzstan (Issyk-Kul Region), eastern Kazakhstan (East Kazakhstan, Jetisu, and Almaty regions), Russia (southern Altai Krai, Altai Republic, Republic of Khakassia, Republic of Tuva, southern Krasnoyarsk Krai), and northwestern China (Xinjiang). It grows on grassy subalpine and alpine meadows, among shrubs and in shady places, on hillsides or at forest edges, and along mountain streams at elevations of 850–2200 m.

Chromosome number: 2*n* = 16 [58,60].

**Figure 8:**
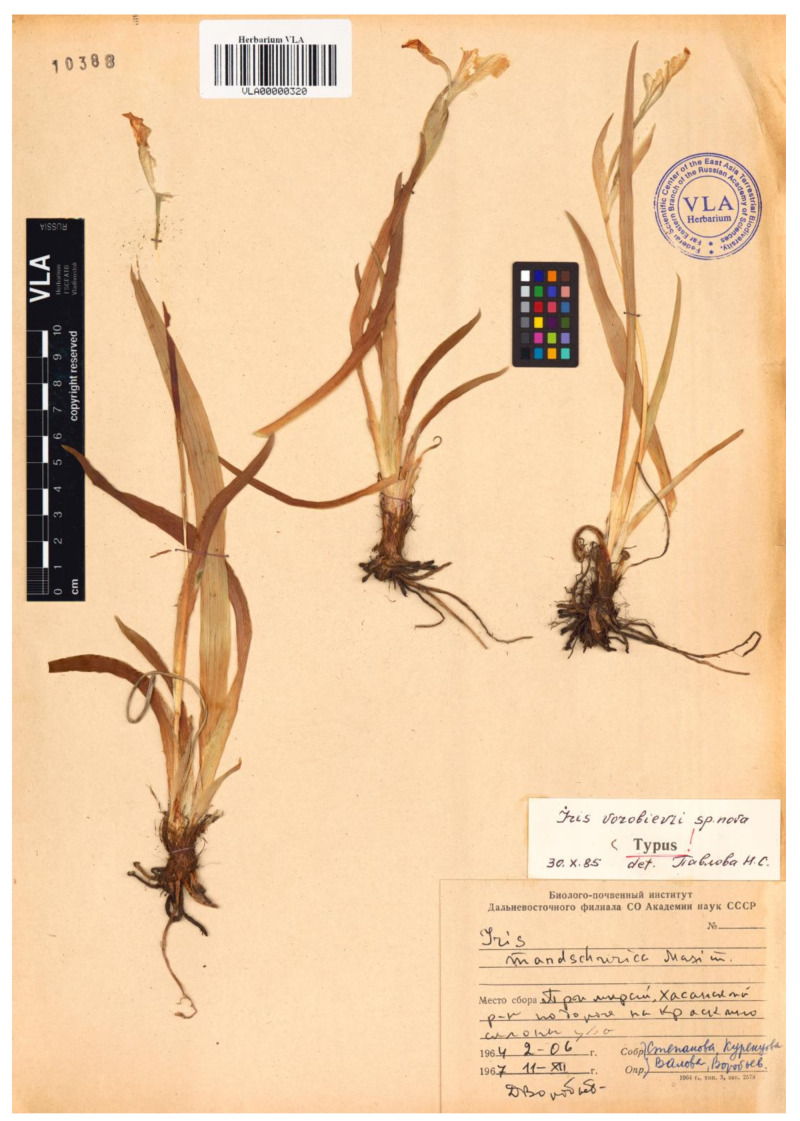
Holotype of *Iris vorobievii* (VLA00000320) (included with permission of the curator).

(3) ***Iris vorobievii*** N.S.Pavlova, Sosud. Rast. Sovet. Dal’nego Vostoka 2: 424, 1987.—Holotype: [Russia] Primorsky Krai, Khasansky District, on the way to Kraskino Village, hill slopes, [fl.], 2 June 1964, *Stepanova* et al. *s.n*. [originally in Russian] (VLA00000320!; isotype VLA00000319!, sub “*Iris mandshurica* Maxim.” det. D.P. Vorobiev).—Figure 8.

Distribution and habitat: This species is known only from a limited area in southern Russian Far East (southern Khasansky District), northern North Korea, and northeastern China (northeastern Jilin Province). It is found growing in open places with good drainage, on loamy sand soil with gravel on grassy slopes, and on shingly meadow terraces near sea coasts at elevations up to 10 m.

Chromosome number: 2*n* = 14 (sub *I. mandshurica*) [108,109].

(II) ***Iris*** ser. ***Potaninia*** Doronkin, Bot. Zhurn. 75(3): 415, 1990.—Type species: *Iris potaninii* Maxim.

(4) ***Iris potaninii*** Maxim., Bull. Acad. Imp. Sci. Saint-Pétersbourg 26(3): 528, 1880.—Lectotype (designated by Alexeeva [116] (p. 417)): [Russia, Irkutsk Oblast] Dahuria, [fl.], 1830, [*Turczaninow*] *s.n*. Herb. C.F. Ledebour (LE01010785!, sub “*Iris flavisima* Pall.” det N.S. Turczaninow et “*Iris potaninii* Maxim. n. sp.” det. C.J. Maximowicz).—http://re.herbariumle.ru/01010785 (accessed on 20 December 2022).

**Figure 9:**
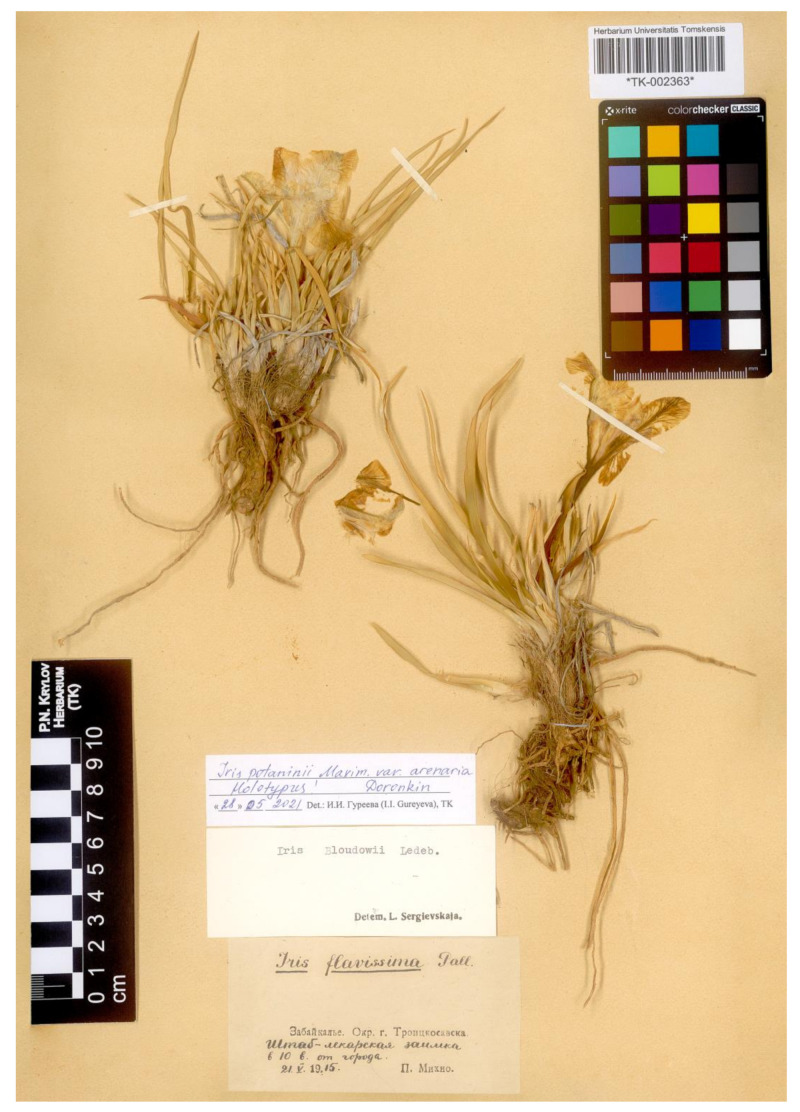
Holotype of *Iris potaninii* var. *arenaria* (TK002363), by permission of the Curator.

= *Iris potaninii* var. *arenaria* Doronkin, Bot. Zhurn. 75(3): 415, 1990.—Holotype: [Russia, Buryatia Republic] Transbaikalia, near Troitskosavsk [Kyakhta], shtab-lekarskaya zaimka, at 10 versts from the city, [fl.], 21 May 1915, *P. Mikhno s.n*. [originally in Russian] (TK002363!, sub “*Iris flavissima* Pall.” det. P.S. Mikhno et “*Iris bloudowii* Ledeb.” det. L.P. Sergievskaya; isotype LE01072716!, sub “*Iris flavissima* Pall.” det. Mikhno et “*Iris potaninii* Maxim.” det. G.I. Rodionenko and V.I. Grubov).—Figure 9.

= *Iris psammocola* Y.T. Zhao, Acta Phytotax. Sin. 30(2): 181, 1992, ***syn. nov***.—Holotype: [China] [Ningxia autonomous region, Lingwu County, Baijiatan], [fl.], 10 April 1959, *s.coll. s.n*. [originally in Chinese] (NENU00014009!).—Figure 7.

= *Iris kamelinii* Alexeeva, Novosti Sist. Vyssh. Rast. 38: 116, 2006, ***syn. nov***.—Holotype: [Russia] Altai Republic, Kosh-Agachsky District, Chikhachev Range, Boguty Lake, the northern gravelly macroslope, 2500 m a.s.l., 6 July 2001, *N.B. Alexeeva* et al. *11* [originally in Russian] (LE01010775!).—http://re.herbariumle.ru/01010775 (accessed on 20 December 2022).

Distribution and habitat: It is distributed in the steppe patches of the southern Siberian mountain systems (Altai Republic, Republic of Khakassia, southern Krasnoyarsk Krai, Republic of Tuva, Republic of Buryatia, Irkutsk Oblast, and Zabaykalsky Krai, Russia), in Mongolia and China (northeastern and western Inner Mongolia, northwestern Heilongjiang Province, and the northern Ningxia autonomous region). The northernmost wild locality of *I. potaninii* known to us has been recorded from the upper Barguzin Depression in the Republic of Buryatia, Russia (54°27′16.5″ N 110°27′08.8″ E; see https://www.inaturalist.org/observations/136986666, accessed on 20 December 2022). As reported in [56], its range covers southern Mongolia from the Gobi-Altai Mountains, Bayankhongor Aimag (e.g., HAL0040724 and HAL0048583; see [103]), and the Gurvan Saikhan Mountains, Ömnögovi Province [118], to western Inner Mongolia and the Ningxia Hui Autonomous Region, China. It often grows in dry rocky, gravelly, or sandy places and on steppe slopes, dunes, and along perennial streams at elevations of 550–2800 m.

Chromosome number: 2*n* = 22 [55,57,58,59,60,61] (55 sub *I. psammocola*; 58,61 sub *I. kamelinii*).

(III) ***Iris*** ser. ***Tigridiae*** Doronkin, Bot. Zhurn. 75(3): 415, 1990.—Type species: *Iris tigridia* Bunge.

(5) ***Iris tigridia*** Bunge, Fl. Altaic. [Ledebour] 1: 60, 1829.—Lectotype (designated here by E.V. Boltenkov): [Russia, Altai Republic] Altai, in schistosis ad fluvium Tscharysch, [fl.], [4 May] 1826, *Bunge 50*, Herb. C.A. Meyer (LE01010797!, sub “*Iris tigridia* Bunge” det. A.A. Bunge).—http://re.herbariumle.ru/01010797 (accessed on 20 December 2022).

= *Iris ivanovae* Doronkin, Fl. Sibir. (Arac.-Orchidac.) 4: 117, 1987.—Holotype: [Russia, Zabaykalsky Krai] Chita Oblast, Borzinskiy District, Kharanor, feather-grass steppe, [fl.], 7 June 1965, *A. Zarubin s.n*. [originally in Russian] (NSK0000077!, sub “*Iris tigridia* Bunge” det. G.A. Peschkova).—https://www.jacq.org/detail.php?ID=525145 (accessed on 20 December 2022).

Distribution and habitat: This species is distributed in southern Siberia, Russia (southern Krasnoyarsk Krai and Republic of Khakassia, southeastern Altai Krai, Altai Republic, Republic of Tuva, southern Republic of Buryatia, and Zabaykalsky Krai), eastern Kazakhstan (East Kazakhstan Region), northern Mongolia, and China (Shanxi, Hebei, Jilin, and Liaoning provinces, Beijing, and Inner Mongolia). It grows in gravelly, stony, or sandy places in steppes among grasses, as well as on dunes, rocky slopes, and often on hilltops at elevations of 400–1200 m.

Chromosome numbers: 2*n* = 38 [58,59]. The other published chromosome numbers are 2*n* = 20, 24, 32, and 40 [57,60], though more studies will be needed to confirm these data.

#### 4.1.2. The Key

Below is a key to the *I*. sect. *Psammiris* species recognized in the present study.

1. Stem > 2.5 cm tall, 1-flowered; perianth tube < 2.5 cm long; flowers of various shades of violet, blue, purple, and lilac . . . *Iris tigridia*

1′. Flowers yellow . . . 2

2. Stem < 2.5 cm tall, 1-flowered; perianth tube > 3.5 cm long . . . *Iris potaninii*

2′. Stem > 3.5 cm tall, several-flowered, simple or branched; perianth tube < 1.8 cm long . . . 3

3. Stem simple (2-flowered), or with 1–2 1-flowered branches; rhizome shortened; roots obconical, storage-like . . . *Iris vorobievii*

3′. Stem simple; rhizome creeping; roots gradually tapering to apex . . . 4

4. Stem with 2–3 flowers and 0–3 bracteoles . . . *Iris humilis*

4′. Stem with 2 flowers and 1 bracteole . . . *Iris bloudowii*

## 5. Conclusions

Although many specialists have carried out extensive studies of *Iris* sect. *Psammiris*, a number of taxonomic problems in this section remain unresolved. Here, we present the first comprehensive molecular phylogeny of the section, with a large set of samples covering most of the distribution ranges and type localities of the species and almost all of its previously recognized taxa. The results obtained in the present study confirm that all previous data, based solely on morphological characters, do not fully clarify the taxonomic composition and phylogenetic relationships among the *I.* sect. *Psammiris* species. Our results based on cpDNA data provide a number of novel insights. The important finding is that the phylogenetic results strongly support the monophyly of *I.* sect. *Psammiris* and the placement of *I. potaninii* var. *ionantha* in the *I*. sect. *Pseudoregelia* clade, which is sister to *I.* sect. *Psammiris*. It should also be emphasized that the taxonomy of *I. potaninii* var. *ionantha* requires further research. Furthermore, the molecular studies confirm the placement of *I. tigridia* in *I*. sect. *Psammiris* rather than in *I*. sect. *Pseudoregelia*.

Other our results are, in general, as follows: (1) five species (*I. arenaria*, *I. humilis*, *I. mandshurica*, *I. pineticola*, and *I. schmakovii*) should be treated as a single species, i.e., *I. humilis*; (2) the specimen listed in reference [55] as *I. psammocola* from Russia and other studied samples from the Tsugeer-Els area (Republic of Tuva, Russia), also referred to as *I. psammocola*, belong to *I. potaninii*; (3) a critical evaluation of the original material and literature showed that *I. psammocola* and *I. potaninii* are the same taxon; (4) the specimens of *I. kamelinii* from the type locality and from Mongolia [17] also belong to *I. potaninii*; (5) the molecular data and a critical examination of the type material and living plants from the type locality confirm that *I. ivanovae*, which has been recognized on the basis of morphology, is a synonym of *I. tigridia*. In view of the findings reported above, we provide an updated classification of *I*. sect. *Psammiris*. The section is unambiguously subdivided into an autonymic series with three species (the most widespread bearded iris *I. humilis*, *I. bloudowii*, and *I. vorobievii*) and two unispecific series: *I*. ser. *Potaninia* with *I. potaninii* and *I*. ser. *Tigridiae* with *I. tigridia*. Thus, here, we present a new taxonomic treatment for *I*. sect. *Psammiris* and an identification key for all of its species. The members of this section are distributed from southeastern Europe through southern Siberia, northern Kazakhstan, China, and Mongolia to the Russian Far East. The results presented herein will undoubtedly contribute to our understanding of the phylogenetic relationships within *Iris* s.l. and the taxonomic composition of the genus in Russia and adjacent areas.

## Figures and Tables

**Table 1:** Sampled *Iris* taxa with voucher information and GenBank accession numbers.

Code (Haplotype)	Locality (Voucher *) **	Coordinates: ° N, ° E	GenBank Accession Nos. *trnH-psbA*/*rps4-trnS*/*trnS-trnG*/*trnL-trnF*
*Iris* subgen. *Iris*			
*I. psammocola* Y.T.Zhao			
TKY (H1)	Russia, Tuva, Kyzyl, *A.Yu. Astashenkov s.n*. (VBGI)	51.58211, 94.35711	ON569443/ON569531/ON569619/ON569707
TTK (H1)	Russia, Tuva, Lake Tore-Khol, *A.Yu. Astashenkov s.n*. (VBGI)	50.15408, 95.13172	ON569444/ON569532/ON569620/ON569708
TTR (H1)	Russia, Tuva, near Tes River, *A.Yu. Astashenkov s.n*. (VBGI)	49.98005, 95.52527	ON569446/ON569534/ON569622/ON569710
TTL (H5)	Russia, Tuva, Tsugeer-Els, *D.N. Shaulo & V.M. Doronkin 17* (LE01072832)	50.333333, 95.484722	ON569445/ON569533/ON569621/ON569709
*I. potaninii* Maxim.			
LBL (H1)	Russia, Altai Republic, Kosh-Agach District, Lake Nizhniye Boguty, *Boltenkov* et al. *38* (VBGI)	49.79277, 89.38888	ON569447/ON569535/ON569623/ON569711
TAS1 (H1)	Russia, Altai Republic, Kosh-Agach District, Tashanta, *Boltenkov* et al. *33* (VBGI)	49.790833, 89.388611	ON569448/ON569536/ON569624/ON569712
TAS2 (H1)	Russia, Altai Republic, Kosh-Agach District, Tashanta, *Boltenkov* et al. *39* (VBGI)	49.76333, 89.2375	ON569449/ON569537/ON569625/ON569713
TAS3 (H1)	Russia, Altai Republic, Kosh-Agach District, Tashanta, *N.V. Shchegoleva s.n*. (VBGI)	49.73307, 89.1562	ON569450/ON569538/ON569626/ON569714
TAS4 (H1)	Russia, Altai Republic, Kosh-Agach District, 6 km north of Tashanta, *P.A. Kosachev* et al. *s.n*. (VBGI)	49.75941, 89.19536	ON569451/ON569539/ON569627/ON569715
SGR (H1)	Russia, Altai Republic, Kosh-Agach District, near the Bol’shoy Sar-Gobo River estuary, *R.V. Kamelin* et al. *2613* (ALTB)	49.66666, 89.09166	ON569452/ON569540/ON569628/ON569716
ARC (H1)	Russia, Altai Republic, Kosh-Agach District, Chagan-Uzun, *Boltenkov* et al. *24* (VBGI)	50.06055, 88.29222	ON569453/ON569541/ON569629/ON569717
ARU (H1)	Russia, Altai Republic, Kosh-Agach District, Chagan-Uzun, *Boltenkov* et al. *26* (VBGI)	50.0725, 88.41416	ON569454/ON569542/ON569630/ON569718
CKR (H1)	Russia, Altai Republic, Ongudaysky District, confluence of Chuya and Katun rivers, *Boltenkov* et al. *22* (VBGI)	50.39722, 86.67444	ON569458/ON569546/ON569634/ON569722
ARS (H1)	Russia, Altai Republic, Ongudaysky District, Shashikman, *Boltenkov* et al. *19* (VBGI)	50.78583, 86.06361	ON569459/ON569547/ON569635/ON569723
KHA (H2)	Russia, Altai Republic, Ongudaysky District, Khabarovka, *I.M. Krasnoborov 188* (MHA)	50.66666, 86.3	ON569460/ON569548/ON569636/ON569724
ARM (H2)	Russia, Altai Republic, Mohro-Oyuk Pass, *A.S. Revushkin* et al. *s.n.* (LE)	49.91769, 87.7311	ON569461/ON569549/ON569637/ON569725
ACH (H4)	Russia, Altai Republic, Kosh-Agach District, Kyzyl-Chin, *P.A. Kosachev* et al. *s.n*. (VBGI)	50.06021, 88.29927	ON569456/ON569544/ON569632/ON569720
ARK (H4)	Russia, Altai Republic, Kosh-Agach District, 7 km west of Kuray, *P.A. Kosachev* et al. *s.n*. (VBGI)	50.23663, 87.87082	ON569457/ON569545/ON569633/ON569721
BNS (H1)	Russia, Buryatia, Novoselenginsk, *Boltenkov 57* (VBGI)	51.01166, 106.64027	ON569462/ON569550/ON569638/ON569726
BGL (H1)	Russia, Buryatia, northeast of Lake Gusinoye, *Boltenkov 62* (VBGI)	51.21861, 106.51472	ON569463/ON569551/ON569639/ON569727
BSA (H6)	Russia, Buryatia, Sakhuli, *D.G. Chimitov & O.V. Imetkhenova s.n*. (UUH)	54.41666, 110.4	ON569464/ON569552/ON569640/ON569728
ZTLS (H1)	Russia, Zabaykalsky Krai, southern bank of Lake Zun-Torey, *Boltenkov 83* (VBGI)	50.00222, 115.72055	ON569465/ON569553/ON569641/ON569729
ZTLW (H7)	Russia, Zabaykalsky Krai, northwestern bank of Lake Zun-Torey, *Boltenkov 77* (VBGI)	50.12972, 115.70361	ON569466/ON569554/ON569642/ON569730
ZTLN (H7)	Russia, Zabaykalsky Krai, north of Lake Zun-Torey, *Boltenkov 80* (VBGI)	50.1675, 115.81583	ON569467/ON569555/ON569643/ON569731
ZAC (H7)	Russia, Zabaykalsky Krai, Adon-Chelon, *Boltenkov 91* (VBGI)	50.46388, 116.0375	ON569468/ON569556/ON569644/ON569732
MTB (H1)	Mongolia, Tow Aimag, Bayan, *Ch. Sanchir s.n*. (LE)	47.25111, 107.53833	ON569469/ON569557/ON569645/ON569733
MTL (H1)	Mongolia, Bayan-Olgii Aimag, Lake Tolbo, *A.I. Shmakov & M.G. Kutsev s.n*. (ALTB)	48.536658, 90.050327	ON569470/ON569558/ON569646/ON569734
MAT (H1)	Mongolia, Arkhangai Aimag, 20 km south of Tsenkher Sum, *I.A. Gubanov 255* (MW)	47.44527, 101.75027	ON569471/ON569559/ON569647/ON569735
MKS (H3)	Mongolia, Khuvsgul Aimag, 25 km north of Sumber, *A.L. Budantsev* et al. *208* (MW)	49.63333, 100.16694	ON569472/ON569560/ON569648/ON569736
*I. kamelinii* Alexeeva			
ABL (H1)	Russia, Altai Republic, Kosh-Agach District, Lake Verkniye Boguty, *Boltenkov* et al. *34* (VBGI) **	49.70583, 89.51333	ON569455/ON569543/ON569631/ON569719
MAK (H3)	Mongolia, Arkhangai Province, Khorgo Mountain, *N.B. Alexeeva* et al. *6* (LE01071966!)	48.18888, 99.84833	ON569473/ON569561/ON569649/ON569737
MKA (H8)	Mongolia, Khuvsgul Aimag, between Khukhuu and Eg-Uur, *N.B. Alexeeva* et al. *36* (LE01071967!)	50.57861, 100.78388	ON569474/ON569562/ON569650/ON569738
*I. bloudowii* Ledeb.			
KIT (H15)	Kyrgyzstan, Issyk-Kul Region, northern slope of Terskey Ala-Too, *A. Naumenko s.n.* (VBGI)	42.676717, 79.167452	ON569475/ON569563/ON569651/ON569739
KAD (H15)	Kazakhstan, Almaty Region, Dzhungraian Alatau, 10 km west of Qapal, *A.V. Grebenjuk 161* (LE)	45.02486, 78.94919	ON569476/ON569564/ON569652/ON569740
AUY (H15)	Russia, Altai Republic, Ust-Kansky District, Yaboganskiy Pass, *Boltenkov* et al. *15* (VBGI) **	50.85194, 85.24194	ON569477/ON569565/ON569653/ON569741
ASH (H15)	Russia, Altai Republic, Shebalinsky Districtn, Shebalino, *Boltenkov* et al. *7* (VBGI)	51.31611, 85.67972	ON569478/ON569566/ON569654/ON569742
ASP (H15)	Russia, Altai Republic, Ongudaysky District, ascent to the Seminsky Pass, *Boltenkov* et al. *40* (VBGI)	50.94472, 85.74111	ON569479/ON569567/ON569655/ON569743
SHA (H15)	Russia, Altai Republic, Ongudaysky District, Shashikman, *L. Lamanova s.n.* (LE)	50.7916, 86.05772	ON569480/ON569568/ON569656/ON569744
AAR (H15)	Russia, Altai Republic, Ongudaysky District, near the Aygulak River estuary, *P.A. Kosachev* et al. *s.n*. (VBGI)	50.35986, 87.24423	ON569481/ON569569/ON569657/ON569745
AKH (H15)	Russia, Altai Republic, Ongudaysky Districtn, Khabarovka, *Boltenkov* et al. *20* (VBGI)	50.66388, 86.29305	ON569482/ON569570/ON569658/ON569746
ACT (H15)	Russia, Altai Republic, Ongudaysky District, Chike-Taman Pass, *Boltenkov* et al. *21* (VBGI)	50.64388, 86.31083	ON569483/ON569571/ON569659/ON569747
AKA (H15)	Russia, Altai Republic, Kosh-Agach District, Aktash, *A. Dedov s.n*. (VBGI)	50.31111, 87.59916	ON569484/ON569572/ON569660/ON569748
*I. pineticola* Klokov			
UPO (H9)	Ukraine, Poltava Oblast, Deimanivka, “Kukvyn”, pine forest, *S.L. Zygalova* et al. *s.n*. (KW) **	50.21666, 32.63388	ON569485/ON569573/ON569661/ON569749
UCH (H9)	Ukraine, Cherkasy Oblast, Irdyn, pine forest, *S.L. Zygalova* et al. *s.n*. (KW) **	49.36916, 31.67916	ON569486/ON569574/ON569662/ON569750
*I. humilis* Georgi			
BOU (H9)	Russia, Belgorod Oblast, west of Urazovo, *s. coll. s.n*. (MHA)	50.07861, 38.04805	ON569487/ON569575/ON569663/ON569751
CHU (H9)	Russia, Altai Krai, Bayevsky District, Chumanka, *A. Dedov s.n*. (VBGI)	53.5, 80.45	ON569496/ON569584/ON569672/ON569760
AAB (H10)	Russia, Altai Republic, Ongudaysky District, Ak-Boom, *N.V. Shchegoleva s.n*. (VBGI)	50.21429, 87.32491	ON569504/ON569592/ON569680/ON569768
CCK (H10)	Russia, Altai Republic, Ongudaysky District, confluence of Chuya and Katun rivers, *Boltenkov* et al. *22* (VBGI)	50.39722, 86.67444	ON569505/ON569593/ON569681/ON569769
ABR (H10)	Russia, Altai Republic, Ongudaysky District, Ak-Boom Rock, *Boltenkov* et al. *23* (VBGI)	50.35361, 87.05694	ON569506/ON569594/ON569682/ON569770
AKC (H10)	Russia, Altai Republic, Kosh-Agach Districtn, between Kurai and Chagan-Uzun, *Boltenkov* et al. *28* (VBGI)	50.16944, 88.20861	ON569507/ON569595/ON569683/ON569771
ATR (H10)	Russia, Altai Republic, Kosh-Agach District, 1 km west of the Tydtugem River estuary, *P.A. Kosachev* et al. *s.n*. (VBGI)	50.18732, 88.12405	ON569508/ON569596/ON569684/ON569772
TTD (H9)	Russia, Tuva, Tandinsky District, Lake Dus-Khol’, *Yu.S. Otmakhov 8* (VBGI)	51.35563, 94.45398	ON569497/ON569585/ON569673/ON569761
TDL (H9)	Russia, Tuva, Lake Dus-Khol’, *Yu.S. Otmakhov 40* (VBGI)	51.35604, 94.44693	ON569498/ON569586/ON569674/ON569762
BSL (H9)	Russia, Buryatia, Tarbagataysky District, Mount Spyashchiy Lev, *Boltenkov 63* (VBGI)	51.53833, 107.34611	ON569491/ON569579/ON569667/ON569755
BBB (H9)	Russia, Buryatia, Bichursky District, Bichura, *Boltenkov 112* (VBGI)	50.62888, 107.66472	ON569492/ON569580/ON569668/ON569756
BTM (H9)	Russia, Buryatia, Tunkinsky District, Mondy, *D.V. Sandanov s.n*. (VBGI)	51.69760, 100.86766	ON569493/ON569581/ON569669/ON569757
BCR (H11)	Russia, Buryatia, Kyakhtinsky District, Chikoy River, Khilgantuy, *Boltenkov 114* (VBGI) **	50.44944, 106.91	ON569494/ON569582/ON569670/ON569758
ZIO (H9)	Russia, Zabaykalsky Krai, Ingoda River, Orlenok, *E.V. Boltenkov 109* (VBGI) **	51.74722, 113.84638	ON569488/ON569576/ON569664/ON569752
ZIV (H9)	Russia, Zabaykalsky Krai, Ingoda River, Ingoda Village, *Boltenkov 110* (VBGI) **	51.83055, 113.08638	ON569489/ON569577/ON569665/ON569753
ZIL (H9)	Russia, Zabaykalsky Krai, Ingoda River, Lesnoi Gorodok, *Boltenkov 111* (VBGI) **	51.66722, 112.98166	ON569490/ON569578/ON569666/ON569754
AMH (H9)	Russia, Amur Oblast, Novotroitskoe Village, *G.F. Darman s.n*. (VBGI)	50.428889, 127.549806	ON569495/ON569583/ON569671/ON569759
ALT-03	Russia, Altai Republic, Ongudaysky District, Chuya River estuary, *L.M. Pshennikova s.n.* (VBGI, cult.)	–	*FM253737/FM253420/FM864187/FM863912*
*I. arenaria* Waldst. et Kit.			
HGY (H12)	Hungary, Győrszentiván, 24.04.2020, *A. Mesterházy s.n.* (VBGI) **	47.69777, 17.73638	ON569501/ON569589/ON569677/ON569765
HCS (H12)	Hungary, Csákvár, *A. Mesterházy s.n*. (VBGI) **	47.39332, 18.46049	ON569502/ON569590/ON569678/ON569766
HBU (H13)	Hungary, Bugac, 28.07.2020, *B. Zoltán s.n*. (VBGI) **	46.65944, 19.59880	ON569503/ON569591/ON569679/ON569767
*I. schmakovii* Alexeeva			
MKK (H9)	Mongolia, Khuvsgul Aimag, Khatgal Sum, *Sh. Baasanmunkh s.n.* (VBGI) **	50.61924, 100.51207	ON569499/ON569587/ON569675/ON569763
MKH (H11)	Mongolia, Khuvsgul Aimag, Lake Khuvsgul, *R.V. Kamelin* et al. *23* (LE) **	50.56666, 100.46666	ON569500/ON569588/ON569676/ON569764
*I. mandshurica* Maxim.			
GSS (H14)	Russia, Primorsky Krai, Oktyabrsky District, Mount Sen’kina Shapka, *Boltenkov s.n.* (VBGI) **	43.91833, 131.65943	ON569510/ON569598/ON569686/ON569774
SRS (H14)	Russia, Primorsky Krai, Oktyabrsky Districtn, Sinel’nikovo-1, *Boltenkov s.n.* (VBGI) **	43.96, 131.53361	ON569511/ON569599/ON569687/ON569775
GSM (H14)	Russia, Primorsky Krai, Nakhodka, Mount Sestra, *Boltenkov s.n.* (VBGI)	42.82777, 132.99499	ON569509/ON569597/ON569685/ON569773
PPE (H14)	Russia, Primorsky Krai, Partizansky District, Ekaterinovka, *Boltenkov 123* (VBGI)	42.91527, 133.04944	ON569512/ON569600/ON569688/ON569776
NAKH-01 NAKH-04	Russia, Primorsky Krai, vicinities of Nakhodka, *R.V. Dudkin s.n.* (VBGI, cult.)	–	*FM253719/FM253402/FM864169/* *FM863894* *FM253722/FM253405/ FM864173/FM863897*
NAKH-07			*FM253725/FM253408/FM864175/* *FM863900*
*I. vorobievii* N.S.Pavlova			
KKR (H16)	Russia, Primorsky Krai, Khasansky District, Kraskino, *Boltenkov s.n.* (VBGI) **	42.725, 130.93361	ON569514/ON569602/ON569690/ON569778
KBR (H16)	Russia, Primorsky Krai, Khasansky District, Bay Pemzovaya, *E.A. Chubar s.n*. (VBGI)	42.54667, 130.83971	ON569513/ON569601/ON569689/ON569777
KRAS-01	Russia, Primorsky Krai, Khasansky District, Kraskino, *R.V. Dudkin s.n.* (VBGI, cult.) **	–	*FM253702 /FM253385/* *FM864152/* *FM863877*
KRAS-04			*FM253705* */FM253388/* *FM864155/FM863880*
KRAS-07			*FM253708/FM253391/* *FM864158/FM863883*
*I. tigridia* Bunge			
ACR (H17)	Russia, Altai Republic, Ust-Kansky District, right bank of Charysh River, Vladimirovka, *Boltenkov* et al. *11* (VBGI) **	51.05388, 84.19	ON569515/ON569603/ON569691/ON569779
AUT (H17)	Russia, Altai Republic, Ust-Kansky District, 3 km west of Tiudrala, *Boltenkov* et al. *10* (VBGI)	51.01, 84.44138	ON569516/ON569604/ON569692/ON569780
ARY (H17)	Russia, Altai Republic, Ongudaysky District, 12 km west of Yelo, *Boltenkov* et al. *17* (VBGI)	50.79055, 85.35777	ON569517/ON569605/ON569693/ON569781
AUS (H17)	Russia, Altai Republic, Ust-Kansky District, east of Ust-Kan, *Boltenkov* et al. *13* (VBGI)	50.94722, 84.82944	ON569518/ON569606/ON569694/ON569782
*I. ivanovae* Doronkin			
BNV (H17)	Russia, Buryatia, Novoselenginsk Village, *Boltenkov 58* (VBGI)	51.01166, 106.64027	ON569523/ON569611/ON569699/ON569787
BAZ (H18)	Russia, Buryatia, Kyakhtinsky District, shtab-lekarskaya zaimka, *Boltenkov 59* (VBGI)	50.38027, 106.55861	ON569524/ON569612/ON569700/ON569788
BMK (H18)	Russia, Buryatia, Maly Kunaley Village, *Boltenkov 113* (VBGI)	50.61361, 107.83111	ON569525/ON569613/ON569701/ON569789
ZKL (H17)	Russia, Zabaykalsky Krai, Aginsky District, Lake Khaptsagaytuy, *Boltenkov 72* (VBGI)	50.6167, 114.88777	ON569520/ON569608/ON569696/ON569784
ZKV (H18)	Russia, Zabaykalsky Krai, Kharanor Village, *Boltenkov 93* (VBGI) **	50.04666, 116.8225	ON569521/ON569609/ON569697/ON569785
ZBT (H18)	Russia, Zabaykalsky Krai, Bol’shaya Tura Village, *Boltenkov 66* (VBGI)	51.63111, 114.0383	ON569519/ON569607/ON569695/ON569783
ZSM (H18)	Russia, Zabaykalsky Krai, Soktui-Milozan Village, *Boltenkov 102* (VBGI)	50.09916, 117.85861	ON569522/ON569610/ON569698/ON569786
*I. goniocarpa* Baker			
CSS	China, Sichuan, Songpang County, Shuijing Village, *T.G. Elumeeva s.n*. (MW0735242)	32.98123, 103.68576	ON569526/ON569614/ON569702/ON569790
CGJ	China, Gansu, Jonê County, Wanmaoxiang, *SQAE 85* (E)	34.8013, 103.20255	ON569527/ON569615/ON569703/ON569791
*I. potaninii* var. *ionantha* Y.T.Zhao			
CQX	China, Qinghai, Xinghai County, northern slope of Jiangluling, *D.G. Long* et al. *148* (E00141064!)	35.56576, 99.98481	ON569530/ON569618/ON569706/ON569794
*Iris* sp.			
CQM	China, Qinghai, Madoi County, Heihe Town, *M. Sun s.n*. (NENU)	34.797384, 98.133337	ON569528/ON569616/ON569704/ON569792
CSJ	China, Sichuan, Jiulong County, Tributary valley SW of Jiulong – Wuxuhai road, *s.coll. 342* (E00424870!)	28.96863, 101.40036	ON569529/ON569617/ON569705/ON569793
Outgroup specimens			
*I.* subgen. *Pardanthopsis* (Hance) Baker			
*I. dichotoma* Pall.	Russia, Amur Oblast, *M. Baranova s.n*. (LE, cult.)	–	*LT978555/LT981297/LT984447/LT984483*
*I.* subgen. *Limniris* (Tausch) Spach			
*I.* ser. *Lacteae* Doronkin			
*I. lactea* Pall.	Russia, Zabaykalsky Krai, Kharanor, *Chernova s.n*. (IRK)	–	*LT627854/LN871708/LN871662/LN871625*
*I. oxypetala* Bunge	China, Shaanxi, Suyde, *Kabanov s.n*. (LE)	–	*LT627844/LT627950/LT627975/LT627911*
*I. tibetica* (Dykes) Bolt.	China, Qinghai, Xining to Ta Er, *Long* et al. *3* (E)	–	*LT627893/LT627939/LT627998/LT627933*
*I.* ser. *Laevigatae* (Diels) G.H.M.Lawr.			
*I. ensata* Thunb.	Russia, Primorsky Krai, Zarubino, *Boltenkov s.n*. (VBGI)	–	*LT628002/LT628022/LT628012/LT627896*
*I. laevigata* Fisch.	Russia, Primorsky Krai, Roshchino, *Pshennikova s.n*. (VBGI)	–	*LT628003/LT628024/LT628013/LT627897*
*I. pseudacorus* L.	Russia, Vladivostok, *Boltenkov s.n*. (VBGI)	–	*LT628004/LT628025/LT628014/LT627898*
*I*. ser. *Ruthenicae* (Diels) G.H.M.Lawr.			
*I. uniflora*	Russian Federation, Primorsky Krai, Zarubino, *Boltenkov s.n.* (VBGI)	–	*LT628002/LT628022/LT628012/LT627896*
*I.* ser*. Sibiricae* (Diels) G.H.M.Lawr.			
*I. sibirica* L.	Mongolia, Dornod, Bayan-Uul, *Gubanov 550* (MW)	–	*LT978556/LT981298/LT984448/LT984480*
*I. bulleyana* Dykes	China, Yunnan, Zhongdian, *M.G. Pimenov* et al. *432* (MW)	–	*LT627895/LT628011/LT628021/LT628001*
*I. bulleyana* f. *chrysographes* (Dykes) Bolt.	China, Sichuan, Jiulong, *Sichuan* *Expedition 331* (E)	–	*LR597328/LR597344/LR597360/LR597376*
*I. bulleyana* f. *forrestii* (Dykes) Bolt.	China, Yunnan, Lijiang, Yulong Xueshan, *P. Cox* et al. *2633* (E, cult.)	–	*LT978553/LT981295/LT984445/LT984478*
*I. delavayi* Micheli	China, Yunnan, Dali Xian, Yinglofen, *Sino-Amer. Bot. Expedition 959* (MHA)	–	*LT978552/LT981294/LT984444/LT984477*
*I. clarkei* Baker ex Hook.f.	Nepal, Trogsindho Pass, *E.F. Needham 674* (E, cult.)	–	*LR597338/LR597354/LR597370/LR597386*
*I. wilsonii* C.H.Wright	China, Yunnan, Little Zhongdian, *E.J. Cowley 566* (Kew no. 1990-3457, cult.)	–	*LR597339/LR597355/LR597371/LR597387*

* Herbarium codes are according to *Index Herbariorum* [83]. ** Specimen collected in/near the type locality. A dash (“–”) indicates that data were not provided. The accession numbers highlighted in italics are reported in references [34,80,81,82]. Cult., cultivated.

**Table 2:** Morphological characteristics of the *Iris* sect. *Psammiris* species.

No.	Character	*I. humilis*	*I. bloudowii*	*I. vorobievii*	*I. potaninii*	*I. tigridia*
1	Rhizome shape	Creeping	Creeping	Shortened	Compact	Creeping
2	Rhizome diameter	0.35–0.8	0.4–1	0.5–1	0.45–1	0.25–0.65
3	Root shape	Equal	Equal	Obconical	Contractile	Contractile
4	Root diameter	0.05–0.2	0.06–0.24	0.12–0.27	0.07–0.34	0.12–0.44
5	Leaf shape	Ensiform or subfalcate	Subfalcate	Subfalcate	Ensiform	Ensiform or subfalcate
6	Leaf apex	Acute, straight or incurved	Acute, incurved	Acute, incurved or straight	Narrowly acute, straight or incurved	Narrowly acute or acute, straight
7	Leaf texture	Thin, smooth	Thin, smooth	Thin, ribbed	Tough, smooth	Tough, smooth
8	Leaf length	10.5–30	21–50	19–60	5–29	9.5–30
9	Leaf width	0.2–1.7	0.4–1.8	0.5–1.7	0.1–0.5	0.1–0.6
10	Stem height	2–19.5	6–28.5	6–25	0.5–2.5	2.5–20
11	Stem branching	0	0	0–2	0	0
12	Number of flowers	2–3	1–2	2–4	1	1
13	Cauline leaf length	4.3–14	11–19	5.2–13.5	2.5–6	3–10
14	Number of bracteoles	(0) 1–3	1	1	0	0
15	Bract length	2–5.6	2.5–5.6	2.5–6.5	2.5–6	3–4.5
16	Bract texture	Tough	Tough	Tough	Thin	Thin
17	Pedicel length	0.2–3	0.8–6	0.5–1.5	0–0.2	0–0.7
18	Tube length	0.5–1.5	0.9–1.8	0.5–1.6	3.5–5.7	1.5–2.5
19	Flower color	Yellow (white)	Yellow	Light yellow	Yellow	Blue to violet (white)
20	Fruit length	2.7–6.5	3.3–6.5	5.2–6.5	2–4	2–4
21	Fruit width	1–2	1.3–2.5	1.2–2.4	0.8–2	0.8–1.8
22	Fruit shape	Elliptical, tapering at apex	Elliptical	Oblong-elliptical, tapering at apex	Elliptical, apex obtuse	Elliptical, tapering at apex

All measurements are in centimeters. See Supplementary raw data in Table S1 for more details. Descriptions of the characters are provided in Section 2.4; for illustrations, see Figure 1 and Figure 6.

## Data Availability

The sequences resulting from this study are available in the NCBI database (https://www.ncbi.nlm.nih.gov/, accessed on 15 January 2023) with GenBank accession numbers ON569443–ON569794.

## Supplementary Materials

The following supporting information can be downloaded at: https://www.mdpi.com/article/10.3390/plants12061254/s1, Table S1: Raw data of the morphological analysis (the codes of the characters are provided in Abbreviations).

## Abbreviations

The following abbreviations are used in this manuscript. AMOVA, analysis of molecular variance; BI, Bayesian inference method; BL, bract length; BP, bootstrap percentage; CL, cauline leaf length; cpDNA, chloroplast deoxyribonucleic acid; FL, fruit length; *F*_ST_, pairwise genetic distances; FW, fruit width; ICN, *Shenzhen code*; *K*_S_, nucleotide sequence divergence; LL, leaf length; LW, leaf width; MCMC, Markov chain Monte Carlo method; MJ, median-joining method; ML, maximum likelihood method; MP, maximum parsimony method; PCR, polymerase chain reaction; PL, pedicel length; PP, Bayesian posterior probability; RhD, rhizome diameter; RoD, root diameter; SH, stem height; TBR, tree bisection–reconnection.
